# Impact of Regular Intake of Microalgae on Nutrient Supply and Cardiovascular Risk Factors: Results from the NovAL Intervention Study

**DOI:** 10.3390/nu15071645

**Published:** 2023-03-28

**Authors:** Fabian Sandgruber, Anna-Lena Höger, Julia Kunze, Benjamin Schenz, Carola Griehl, Michael Kiehntopf, Kristin Kipp, Julia Kühn, Gabriele I. Stangl, Stefan Lorkowski, Christine Dawczynski

**Affiliations:** 1 Junior Research Group Nutritional Concepts, Institute of Nutritional Sciences, Friedrich Schiller University Jena, Dornburger Str. 29, 07743 Jena, Germany; fabianalexander.sandgruber@uni-jena.de (F.S.); juku16@googlemail.com (J.K.); benjamin.schenz@web.de (B.S.); 2 Competence Cluster for Nutrition and Cardiovascular Health (nutriCARD) Halle-Jena-Leipzig, Dornburger Str. 25, 07743 Jena, Germany; julia.kuehn@landw.uni-halle.de (J.K.); gabriele.stangl@landw.uni-halle.de (G.I.S.); stefan.lorkowski@uni-jena.de (S.L.); 3 Competence Center Algal Biotechnology, Anhalt University of Applied Science, Bernburger Str. 55, 06366 Köthen, Germany; leni.hoeger@gmail.com (A.-L.H.); carola.griehl@hs-anhalt.de (C.G.); 4 Institute of Clinical Chemistry and Laboratory Diagnostics, University Hospital Jena, Am Klinikum 1, 07747 Jena, Germany; michael.kiehntopf@med.uni-jena.de; 5 Department of Pediatrics and Adolescent Medicine, Sophien- and Hufeland Hospital, Henry-Van-De-Velde-Str. 1, 99425 Weimar, Germany; k.kipp@klinikum-weimar.de; 6 Institute of Agricultural and Nutritional Science, Martin Luther University Halle-Wittenberg, Von-Danckelmann-Platz 2, 06120 Halle, Germany; 7 Institute of Nutritional Sciences, Friedrich Schiller University Jena, Dornburger Str. 25, 07743 Jena, Germany

**Keywords:** nutrients, bioavailability, human trial, intervention study, *Microchloropsis*, *Chlorella*, omega-3 fatty acids, vitamin D, vitamin B_12_, uric acid

## Abstract

A 14-day randomized controlled study with a parallel design was conducted with 80 healthy participants. Intervention groups I (IG1) and II (IG2) received a defined background diet and consumed a smoothie enriched with either 15 g of *Chlorella* dry weight (d.w.) or 15 g of *Microchloropsis* d.w. daily. Control group II (CG2) received a defined background diet without the smoothie. Control group I (CG1) received neither. Blood samples and 24-h urine were collected at the beginning and the end of the study. Serum concentrations of 25-hydroxyvitamin D_3_, vitamin D_3_, selenium, iron, ferritin, transferrin saturation, total cholesterol, low-density lipoprotein (LDL) cholesterol, high-density lipoprotein (HDL) cholesterol, non-HDL cholesterol and the LDL-cholesterol/HDL cholesterol ratio decreased in IG1 (*p* < 0.05), while 25-hydroxyvitamin D_2_ increased (*p* < 0.05). In IG2, vitamin D_3_, 25-hydroxyvitamins D_2_ and D_3_ decreased (*p* < 0.05), while concentrations of fatty acids C20:5_n3_ and C22:5_n3_ increased. Serum and urine uric acid increased in IG1 and IG2 (*p* < 0.05). *Microchloropsis* is a valuable source of n3 fatty acids, as is *Chlorella* of vitamin D_2_. Regular consumption of *Chlorella* may affect the iron and selenium status negatively but may impact blood lipids positively. An elevated uric acid concentration in blood and urine following the regular consumption of microalgae poses potential risks for human health.

## 1. Introduction

Microalgae are of interest because they contain carotenoids, vitamins, long-chain (LC) omega-3 (n3) fatty acids, minerals and trace elements and usually have an amino acid profile favorable for human nutrition [1]. However, microalgae are rarely used for food production. Microalgae are considered not a traditional but a novel food in the European Union, and one of the main obstacles is that their authorization for the European food market is strictly regulated by the Novel Food Regulation (EC) no. 258/97.

*Chlorella pyrenoidosa* was identified and named in 1903 based on the presence of pyrenoids in chloroplasts [2]. It has been produced in Taiwan since 1987 and commercially used in Belgium, which excludes *Chlorella pyrenoidosa* from the Novel Food Regulation (EC) no. 258/97 and authorizes it as food or a food ingredient [3]. The unicellular *Chlorella* subspecies is known for its high concentrations of total fiber, proteins, trace elements and vitamin D [1]. *Chlorella*-based products have steadily increased in the food market and are predicted to play an important role in future nutrition [4].

The *Microchloropsis* species is of particular interest due to its high quantities of LC n3 FA and its comparatively simple cultivation that enables mass production [5]. So far, *Microchloropsis salina* is not among the 10 microalgae authorized by the European Commission for human nutrition.

Both microalgae show the most promise for human nutrition due to their high concentrations of various nutrients. The bioavailability of nutrients in microalgae is controversially discussed. Contrary data exist, for example, for the bioavailability of vitamin B_12_ in humans [6,7]. Furthermore, the digestion of microalgae and the bioavailability of their nutrients may depend on the food matrix. Hence, it has been shown that the in vitro digestibility of microalgae and the bioavailability of carotenoids may vary depending on processing, such as cell disruption and lipid content of microalgae [8,9,10].

The regular consumption of various nutrients can prevent diseases such as hypertension, hyperlipidemia and hyperglycemia in humans [11,12,13,14]. Nutrients particularly relevant in this context are LC n3 FA, vitamin D and fibers. Previous studies have shown that the consumption of LC n3 FA, which occur in higher quantities, especially in *Microchloropsis salina*, is associated with a reduction in elevated triglycerides, blood pressure and thrombotic events [15,16]. In addition, adequate concentrations of vitamin D, unlike low concentrations, have been associated with an improvement of the atherogenic lipid profile [17] and hypertension [18]. Similarly, the protective effects of the consumption of dietary fiber are associated with the reduction in several risk factors for cardiovascular diseases and an improvement of the gut microbiome [19,20].

This study investigated the bioavailability of chosen nutrients from *Chlorella pyrenoidosa* and *Microchloropsis salina* for human nutrition. The influence of the regular consumption of both microalgae on human health, especially on cardiovascular risk factors, was also evaluated.

## 2. Materials and Methods

### 2.1. Subjects

Eighty healthy participants (52 females and 28 males) aged between 20 and 35 years and with a body mass index (BMI) of ≤30 kg/m^2^ were enrolled after providing written informed consent. The age range was chosen to reduce possible effects on the studied blood and urine parameters due to hormonal fluctuations, health issues or interference of medications. The exclusion criteria for the participation in the NovAL study for all participants were as follows: acute or chronic disease (cardiovascular disease, tumor, infection), gastrointestinal diseases, diabetes mellitus type 1 and 2, chronic renal disease, diseases of the parathyroid, diseases that require regular phlebotomies and other chronic diseases; use of prescription medicine, which could affect results of the study; intake of lipid-lowering drugs, diabetes medication or hormone replacement therapy; estimated glomerular filtration rate (eGFR) < 60 mL/min; weight loss (≤3 kg) or weight gain (≥3 kg) during the last 3 months before the study began; pregnancy or lactation; transfusion of blood in the last 3 months before blood sampling; use of supplements, including vitamins, fish oil, minerals and trace elements (3 months before and during the entire study period); vegetarians, vegans or food allergies; alcohol and drug abuse; elite athletes (>10 h of strenuous physical activity per week); simultaneous participation in other clinical studies; or the inability (physically or psychologically) to comply with the procedures required by the protocol.

The primary endpoint of the study is the change in eicosapentaenoic acid (EPA) concentration in plasma lipids. This study uses total subject and group sizes based on data from Dawczynski et al. [21]. The EPA concentrations in plasma lipids increased from 0.72 ± 0.35% at the beginning to 1.69 ± 0.94% after the 14-week intervention with LC n3 polyunsaturated fatty acids (PUFA)-supplemented dairy products. Accordingly, a group size of 10 subjects has >95% power. Considering that the envisaged intervention period is shorter the human study from Dawczynski et al., the number of subjects per group was doubled (total subjects = 4 × 20). The power analysis was performed with G*Power version 3.1.9.2 (Heinrich Heine University Düsseldorf, Düsseldorf, Germany).

### 2.2. Study Design

**Figure 1:**
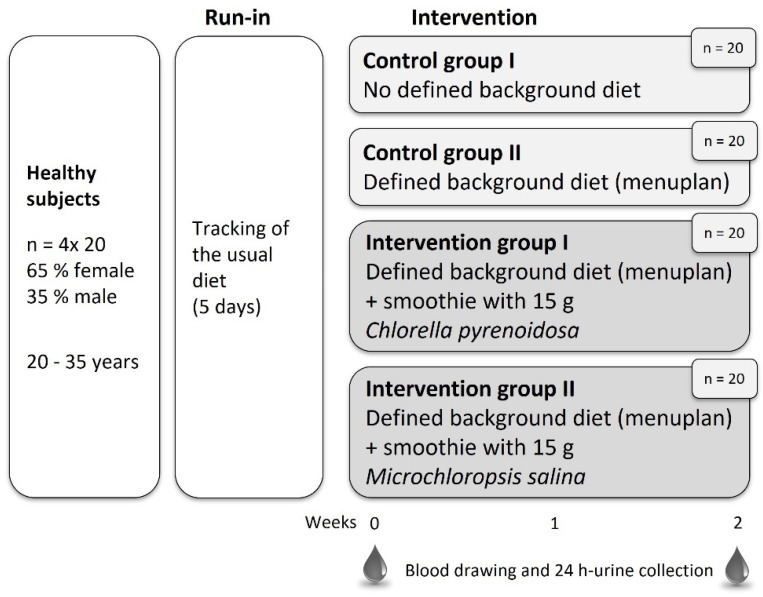
Design of the 4-arm randomized controlled NovAL intervention study.

A randomized controlled study with a parallel design was conducted (Figure 1). The 80 subjects were randomly divided into 4 groups (allocation ratio: 1:1:1:1). The randomization was performed with RandomizerR by R-Studio statistics (RStudio PBC, Boston, MA, USA). Patients in the 2 intervention groups and control group II (CG2) received menu plans to standardize their background diet over the study period. The plans were adapted for energy and nutrient requirements, which in turn were dependent on age, sex and physical activity of the participants. Subjects in both intervention groups consumed a smoothie daily, either with 15 g of *Chlorella pyrenoidosa* d.w. or 15 g of *Microchloropsis salina* d.w. Two control groups that were not given microalgae were included in the study. Control group I (CG1) received no standardized menu plans, whereas CG2 cooked from defined menu plans. In the run-in phase of 5 days, participants had to keep a nutrition diary, and their physical activity was tracked. Collections of blood and 24-h urine samples were obtained at the beginning and the end of the 14-day treatment period. Furthermore, anthropometric data, blood pressure and body composition were determined.

The study was conducted in accordance with the Helsinki Declaration of 1975 as revised in 1983. The study protocol of the NovAL study was reviewed and approved by the Ethical Committee of the Friedrich Schiller University of Jena (no. 2020-1650-BO). The study was registered on https://clinicaltrials.gov/ct2/show/NCT04567823 (accessed on 25 March 2023).

### 2.3. Diet

**Figure 2:**
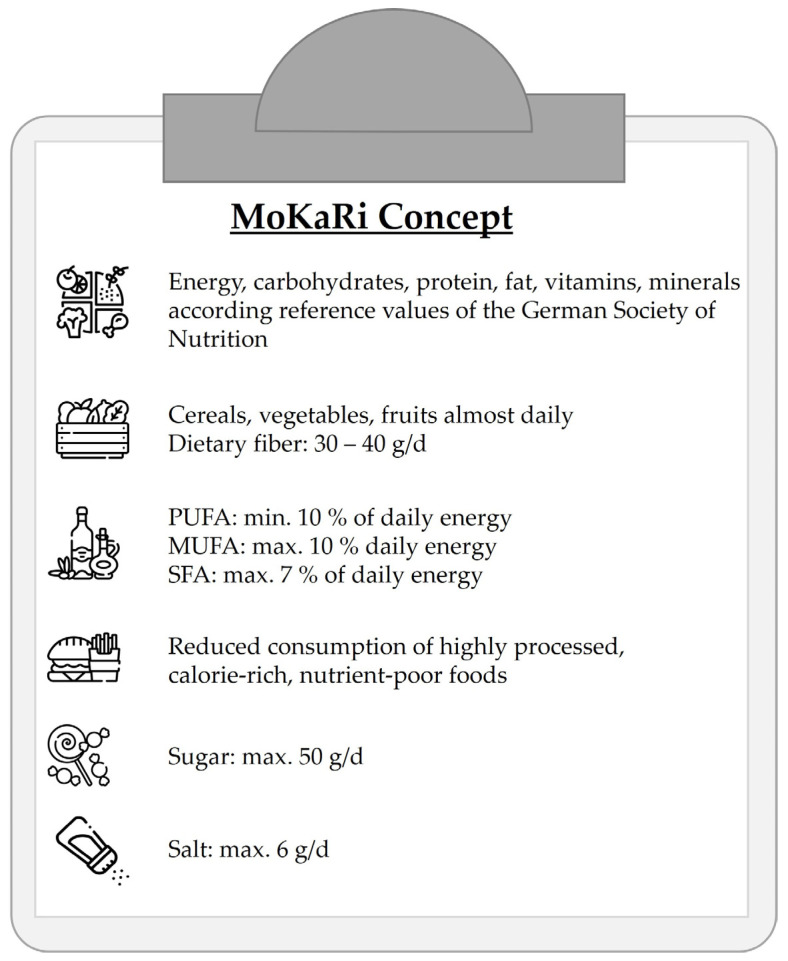
Criteria of the MoKaRi concept (MUFA, monounsaturated fatty acids; PUFA, polyunsaturated fatty acids; SFA, saturated fatty acids).

The participants of CG2 and the intervention groups were given individual menu plans that were developed according to the MoKaRi concept (Figure 2). However, menu plans containing fish and seafood were excluded from the diet, and the total fiber consumption was slightly reduced compared to the MoKaRi concept [22].

### 2.4. Intervention Food Product

The intervention groups received a smoothie daily with either 15 g of *Chlorella* d.w. or 15 g of *Microchloropsis* d.w. This study used microalgae from the Competence Center Algal Biotechnology of Anhalt University of Applied Science in Germany, which was spray-dried and ground with a ball mill. The microalgae powders were tested for microbial contamination. The ingredients of the smoothies (banana, pineapple, kale, mango, dates, avocado, lime juice, wheatgrass, mint and the chosen microalgae) were shock-frozen and stored at −20 °C. During the intervention period, the participants were asked to add 160 mL of water to the intervention product and blend it for 2 min. After blending the ingredients to a smooth, clump-free consistency, the participants consumed the smoothie directly. Afterwards, the blender and the cup containing the smoothie were rinsed out with a predefined amount of water. The nutrient profile of both intervention smoothies is listed in Table 1, and the fatty acid profile is in Table 2. The methods used, instruments and the institutes that analyzed the respective parameters from Table 1 and Table 2 are listed in the Supplemental Materials (Table S1).

### 2.5. Blood, Urine and Body Parameters

Blood samples were collected by venipuncture after a minimum of 10 h of fasting at the beginning and the end of the study period (Figure 1). Plasma and serum parameters were analyzed according to standard operating procedures of the involved laboratories as described in the Supplemental Materials. Urine was collected by the participants for 24 h prior to blood drawing in a 3-liter container. Methods, instruments and references as well as the analytical institutes are listed in the Supplemental Materials (Table S2). The height of the participants was measured with the portable stadiometer 213 (Seca, Hamburg, Germany). Body composition was analyzed by the medical body composition analyzer mBCA 515 (Seca), namely, body water, body fat, lean body mass, extracellular mass, body cell mass and BMI. Systolic and diastolic blood pressure were measured with a sphygmomanometer (Boso Compact S, Bosch + Sohn, Jungingen, Germany).

### 2.6. Lipid Extraction and Fatty Acid Analysis in Total Plasma Lipids

Sample preparation and fatty acid analysis were performed as described [23]. Plasma was gained by centrifugation of collected blood in lithium–heparin monovettes (10 min, 4 °C, 2500× *g*). Fat was extracted according to the procedure of Folch and Bligh and Dyer [24,25]. The extracted lipids were saponified and methylated with NaOCH_3_ and BF_3_ [26]. The resulting fatty acid methyl esters (FAME) were analyzed via a gas chromatograph (GC; GC-17 V3, Shimadzu, Duisburg, Germany) equipped with an AOC-5000 auto-sampler (Shimadzu) and flame ionization detector (Shimadzu). A fused-silica capillary column DB-225 ms (30 × 0.25 mm, i.d. with 0.2 µm film thickness; J and W Scientific, Folsom, CA, USA) was used. The carrier gas was H_2_. For quantification of each FAME, solution software (LabSolution LC/GC release 5.92, Shimadzu) was used. FAME are presented in relation to the total FAME content.

### 2.7. Statistical Analysis

The statistical analyses were conducted using SPSS Statistics Premium version 27 (IBM, Chicago, IL, USA). A *p*-value of <0.05 was considered to display significant changes. The results are presented as medians and interquartile ranges (IQRs). The Shapiro–Wilk test was performed to determine normal distribution. To detect significant differences between the 4 intervention groups, Welch’s ANOVA was used if the residuals showed normal distribution and the Kruskal–Wallis test with a paired Wilcoxon signed-rank test if the normal distribution was denied. For the analysis of significant changes within the same intervention group but between the first and last blood or urine collection, a paired t-test was performed at normal distribution and the Wilcoxon test when normal distribution was not confirmed. In addition, the Benjamini–Hochberg procedure was executed to decrease the false discovery rate of significant changes which might occur because of the analysis of multiple parameters.

## 3. Results

### 3.1. Anthropometric Data, Body Composition, Blood Pressure, Energy and Nutrient Intake

**Figure 3:**
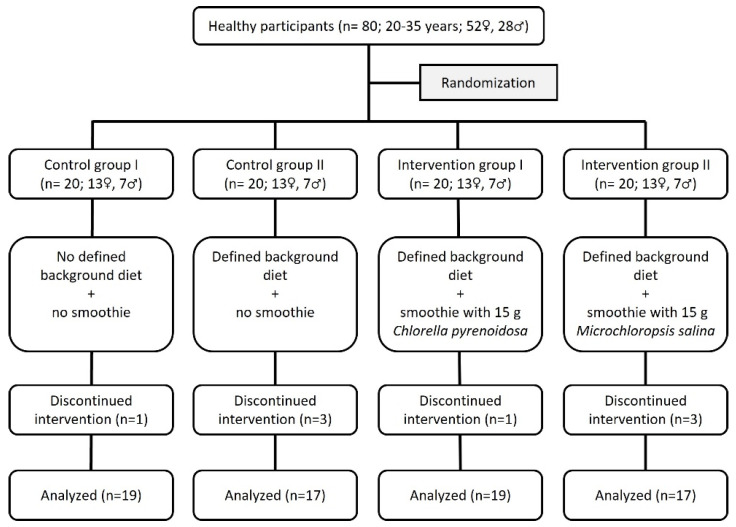
Flow chart of the NovAL study.

Eighty subjects were enrolled in the NovAL study, eight of whom did not fully complete the study due to personal reasons or being unwilling to follow the menu plans (dropout rate: 10%; Figure 3). The participants were randomized into four groups with an average age between 23 and 26 years and BMI between 22.1 and 23.7 kg/m^2^ (Supplementary Table S4). The groups showed no significant differences in age, height, body weight or BMI. The measured parameters of body composition as well as systolic and diastolic blood pressure did not change over the study period (Supplementary Table S4). Energy and nutrient intake in the week before starting the intervention were self-reported by the participants over five days. The energy and nutrient intake were comparable between all four groups except for the intake of ALA, which was higher in CG2 than in CG1 (*p* < 0.05; Supplementary Table S3).

### 3.2. Nutrient Status

The plasma vitamin C concentrations increased compared to baseline in CG1 (+2.6 mg/L), intervention group I (IG1, +1.9 mg/L) and intervention group II (IG2, +3.3 mg/L; *p* < 0.05), but did not differ from each other and CG1. While vitamin D_2_ concentrations were under the limit of quantification (0.5 nmol/L) in all study groups, 25-hydroxyvitamin D_2_ increased in IG1 (*p* < 0.05). 25-Hydroxyvitamin D_2_ increased, and the end values in IG1 were different from all other groups (*p* < 0.05), yet 25-hydroxyvitamin D_3_ and vitamin D_3_ decreased in all four study groups (*p* < 0.05) but without differences between groups. Furthermore, vitamin B_12_ increased from 244 to 281 nmol/L in IG1 (*p* < 0.05; Table 3), yet no differences were detected between groups. In addition, plasma selenium concentration was lowered from 1.48 to 1.35 µmol/L in IG1 (*p* < 0.05; Table 3). Differences between groups were not determined. In IG1, the plasma iron concentration decreased from 21.5 to 16.8 µmol/L and transferrin saturation from 29.4 to 21.8 mmol/L (*p* < 0.05). In addition, ferritin concentrations decreased from 38.0 to 20.2 µg/L (*p* < 0.05). The concentrations of further vitamins, minerals and trace elements were comparable within and between groups (Table 3).

### 3.3. Fatty Acid Profiles in Plasma Lipids

The saturated fatty acid (SFA) concentrations in plasma lipids range between 29 and 30% FAME with C16:0 (20–21%) and C18:0 (6.6–7.0% FAME) as dominant representatives. Monounsaturated fatty acid (MUFA) concentrations range from 24 to 27% FAME, and omega-6 polyunsaturated fatty acids (n6-PUFAs) vary from 37 to 40% FAME. The described fatty acid groups did not change during the study period (Table 4).

The baseline values of omega-3 polyunsaturated fatty acids (n3 PUFAs) of all study groups (2.7–3.5% FAME) did not differ from those at the end of the study (2.7–3.9% FAME). The end values of the n3 PUFA of IG2 (3.86% FAME) were higher than in IG1 (2.95% FAME; *p* < 0.05). The concentration of C20:5 increased during IG2 from 0.59 to 0.88% FAME (*p* < 0.05). End values of IG2 (0.88% FAME) were also higher than CG1 (0.40% FAME), CG2 (0.37% FAME) and IG1 (0.41% FAME; *p* < 0.05). In addition, the difference between baseline and end from IG2 (0.24% FAME) was higher than in CG1 (−0.05% FAME), CG2 (−0.09% FAME) and IG1 (−0.10% FAME; *p* < 0.05; Table 4).

In IG2 (9.97), the n6/n3 PUFA ratio was lower after the two-week period than in CG1 (14.14) and IG1 (13.70; *p* < 0.05; Table 4).

### 3.4. Liver and Kidney Function

In all four groups, the activities of alanine aminotransferase, aspartate aminotransferase, γ-glutamyl-transferase and lactate dehydrogenase remained unchanged during the study period (Table 5). Due to IG1, the activity of cholinesterase decreased by 6 µmol/L*s (*p* < 0.05). The change in cholinesterase activity in IG1 differs from that in CG2 (*p* < 0.05; Table 5).

### 3.5. Clotting

The activated partial thromboplastin time, quick value and international normalized ratio remained similarly unchanged during the study (Table 5). The baseline values, endpoints and their respective delta values did not differ between the groups. Fibrinogen concentrations increased by 0.2 nmol/L in IG1 (*p* < 0.05). No differences were detected in the other groups or between all four groups (Table 5).

### 3.6. Blood Count

The baseline and end values of hematocrit, mean corpuscular hemoglobin (MCH), corpuscular hemoglobin concentration (MCHC) and red cell distribution width did not differ between the four groups (Table 5). Mean corpuscular volume (MCV) decreased in both intervention groups by 1 fl (*p* < 0.05). The decrease in MCV in IG2 differed significantly from IG1, CG1 and CG2 (*p* < 0.05).

### 3.7. Cardiovascular Risk Factors

After the intervention period, the total cholesterol concentration decreased in IG1 by 0.82 mmol/L and differed from all other groups (*p* < 0.05). In addition, the end value of total cholesterol in IG1 (3.45 mmol/L) was lower compared to that in CG1 (4.25 mmol/L). Furthermore, high-density lipoprotein (HDL) cholesterol and LDL cholesterol decreased in IG1 by 0.17 and 0.71 mmol/L, respectively. The decrease in LDL cholesterol differed from CG1 (*p* < 0.05). The end values of LDL cholesterol from all menu-plan-receiving groups were lower compared to CG1. The LDL-cholesterol/HDL-cholesterol ratio decreased in IG1 by 0.23 and differed from CG1, where it increased by 0.07 (*p* < 0.05). The end value of the LDL-cholesterol/HDL-cholesterol ratio in CG2 (1.07) was different from CG1 (1.62; *p* < 0.05). In IG1, the non-HDL-cholesterol concentrations decreased after intervention by 0.23 mmol/L and were different from CG1 with an increase of 0.05 mmol/L (*p* < 0.05). The endpoint values of non-HDL cholesterol in IG1 (2.03 mmol/L) were lower than in CG1 (2.80 mmol/L).

Concentrations of uric acid in blood and urine of both microalgae-receiving groups increased due to the intervention (*p* < 0.05; Table 6). However, differences between the groups were not detected. Other parameters reflecting the cardiovascular risk did not differ (Table 6).

### 3.8. Diabetes Risk Factors

The baseline and final plasma concentrations of fasting glucose and insulin did not differ in the four groups (Table 6). Hemoglobin A1c (HbA_1c_) decreased during the course of IG1 (*p* < 0.05). There were no differences in fasting glucose, insulin or HbA_1c_ between the groups.

## 4. Discussion

A review in 1991 highlighted microalgae as a valuable nutrient source [27]. It was further assumed that the consumption of microalgae and their ingredients may prevent diseases such as cardiovascular diseases [28]. Cardiovascular diseases are the leading cause of death in Germany. In 2020, for example, 34% of all deaths in Germany were traced to cardiovascular diseases [29]. Dietary approaches and lifestyle interventions are effective measures in preventing cardiovascular diseases [30]. Therefore, the NovAL study sought to determine the bioavailability of selected nutrients from microalgae and their influence on nutrient status and cardiovascular risk factors.

The nutrient profile of *Chlorella pyrenoidosa* was characterized by high contents of total fiber, protein and vitamins D_2_ and D_3_, whereas the *Microchloropsis salina* profile showed valuable contents of LC n3 PUFA, especially EPA, minerals and trace elements such as zinc, nickel and copper.

### 4.1. Bioavailability of Nutrients

This study found that compared to baseline values in IG1 (*Chlorella pyrenoidosa* d.w.), concentrations of 25-hydroxyvitamin D_3_, vitamin D_3_, selenium, iron, ferritin, MCV and transferrin saturation decreased, while those of vitamin B_12_, C, 25-hydroxyvitamin D_2_, and fatty acids C20:0, C18:1_n9_ and C18:1_n7_ increased. The increase and end values of 25-hydroxyvitamin D_2_ were different from all other groups.

In IG2 (*Microchloropsis salina* d.w.), concentrations of 25-hydroxyvitamin D_3_ and vitamin D_3_ decreased, while those of C17:0, C18:1_n7_, C20:5_n3_, C22:5_n3_, vitamin C and MCV increased. Another interesting finding was that there were lower concentrations of C14:0 compared to CG1 and C20:3_n6_ compared to CG1 and CG2 after the intervention. C20:5_n3_ and C22:5_n3_ concentrations were higher than in all other study groups. n3 PUFA and LC n3 PUFA were higher than in IG1 and the n6/n3 PUFA ratio than in CG1 and IG1.

Surprisingly, the consumption of the *Chlorella* smoothie, while providing 1.65 µg of selenium and 15.9 mg of iron, was related to a decrease in selenium and parameters reflecting iron status (ferritin, iron, transferrin saturation and MCV). Selenium, mainly occurring as selenomethionine in *Chlorella sorokiniana*, appeared to have good bioavailability in the in vitro and in vivo models [31]. Furthermore, various studies have highlighted the potential of microalgae as a plant-based iron source [32,33,34,35]. However, the bioavailability of trace elements, such as iron and selenium, can be decreased not only by polysaccharides but also flavonoids [36,37,38,39].

The concept of the MoKaRi diet used in this study is marked by a daily intake of 30 to 40 g of fiber and smoothies enriched with *Chlorella pyrenoidosa*, which provides an additional 10.4 g of total fiber per serving. However, digestion and bioavailability of nutrients is inhibited by the robust cell wall of *Chlorella* [40]. On the other hand, high contents of Fe and Zn seem to have an additional enhancing effect on the absorption of selenium [41,42]. The bioavailability of selenium and iron might have been affected by those factors.

Vitamin B_12_ is distinguished into its bioavailable form cobalamin and its biologically nonactive form pseudo-vitamin B_12_ resulting from the α-ligand binding the cobalt in the center of the corrin ring [43]. Previous studies indicate primarily the enrichment of pseudo-vitamin B_12_ in microalgae [44,45]. In the present study, the analyzed contents of bioactive vitamin B_12_ in both microalgae were under 0.3 µg/100 g. In 2003, a study determined that 4 to 406 µg of bioavailable vitamin B_12_ and less than 44 µg of pseudo-vitamin B_12_ were present in the dry weight of various *Chlorella pyrenoidosa* supplements, establishing *Chlorella pyrenoidosa* as a viable source of vitamin B_12_ [46]. In our study, the vitamin B_12_ concentrations in the serum increased in the *Chlorella*-receiving group of the NovAL study participants. We assume that there might indeed be bioavailable amounts of vitamin B_12_ in the *Chlorella* powder used in the study. The small and insignificant increase in holo-transcobalamin in the serum supports this hypothesis. Previous studies on vegetarians and vegans with vitamin B_12_ deficiency have shown that the participants could improve their vitamin B_12_ status after supplementation of 9 g/d of *Chlorella pyrenoidosa* over a period of 60 days [6]. An improvement in vitamin B_12_ status was also achieved in deficient Wistar rats after 13 weeks with a 4 to 8% *Chlorella*-containing diet [6]. Hence, it cannot be ruled out that *Chlorella* is a bioavailable vitamin B_12_ source for humans.

Vitamin D_2_ is mainly found in fungi and yeast, which synthesize vitamin D_2_ by UVB exposure [47]. The vitamin D_2_ concentration in *Chlorella* is probably caused by the symbiotic cultivation of *Chlorella* and yeasts that are able to synthesize vitamin D_2_. This symbiotic cultivation is a common method to increase protein and lipid production in *Chlorella pyrenoidosa* by using the monosaccharides from yeast [48]. Research in this area has highlighted the potential of microalgae as a vitamin D source [49]. The intake of vitamin D_2_ by *Chlorella pyrenoidosa* (63 µg per smoothie) was followed by an increase in serum 25-hydroxyvitamin D_2_ concentrations. However, the amounts absorbed were too low to counteract the decrease in 25-hydroxyvitamin D_3_, which is the result of a reduced synthesis of vitamin D due to less UVB exposure in winter [50]. To our knowledge, there are no human trials on the bioavailability of vitamin D from microalgae available. Similar results, on the other hand, were reported for the consumption of vitamin D_2_-enriched wheat germ oil, which increased plasma 25-hydroxyvitamin D_2_ concentrations but could not prevent vitamin D_3_ reduction. Furthermore, this study indicated a disproportionate reduction in 25-hydroxyvitamin D_3_ by vitamin D_2_ absorption [51]. These effects were not determined in our study. However, it cannot be ruled out that the consumption of higher amounts of *Chlorella* would improve general vitamin D status.

A good bioavailability of EPA was detected after daily consumption of 0.7 g of EPA from *Microchloropsis salina,* which resulted in higher EPA and docosapentaenoic acid (DPA, C22:5_n3_) concentrations in the plasma compared to all other study groups. Increased LC n3 PUFA intake, mostly due to the consumption of LC PUFA-containing fish, is associated with lower incidences of cardiovascular diseases [15,52,53]. Eicosanoids such as prostaglandins, thromboxanes and leukotrienes synthesized from C20:4_n6_ are important regulators and mediators of inflammatory processes [54]. LC n3 PUFA, such as EPA, inhibit C20:4_n6_ metabolism by inhibiting the induction of cyclooxygenase 2, an enzyme at the beginning of the prostaglandin and thromboxane biosynthesis [55]. Via *elongase,* EPA can be elongated to DPA in humans [56]. DPA has recently gained attention because of its role in inflammatory processes, lipid metabolism and cognitive function [57]. Higher concentrations of circulating DPA in the blood are linked with lower total and cancer mortality as well as mortality from coronary heart diseases [58]. Fish and seafood are common sources of DPA [57]. Previous clinical trials demonstrated an increase in EPA and DPA after supplementation of 6 g of EPA/d for six days and 0.44–2.70 g of EPA/d for 12 weeks [59,60]. These findings, however, are not in line with the data of the NovAL study.

Due to its radical scavenger function and antioxidative property, vitamin C is a key factor for the human immune system [61]. Even though the variety of foods is larger and availability is greater than ever before, a study from Germany in 2018 analyzing the vitamin C status of 300 healthy participants indicated a vitamin C deficit in 17.4% of the attending individuals [62]. The average vitamin C status in three out of four groups of the NovAL cohort was in the normal range of 5 to 15 mg/L for vitamin C in plasma but close to a deficit. The increased consumption of fruits and vegetables according to the adapted menu plans of the MoKaRi concept (averaging 268 mg of vitamin C per day) improved vitamin C status compared to baseline in all groups receiving menu plans. This demonstrates the compliance with the menu plans in the respective groups. The additional consumption of fruits and vegetables from the smoothie further augmented the vitamin C increase.

Despite the very different nutrient profiles of *Chlorella pyrenoidosa* and *Microchloropsis salina*, the nutrients status of the study participants hardly changed compared to the control groups. We assume that a longer duration of the study or the consumption of more than 15 g of microalgae might show better effects on nutrient status. 

### 4.2. Influence on Human Health

In IG1, MCV, fibrinogen, uric acid in blood and urine increased compared to baseline, whereas the concentrations of total cholesterol, LDL cholesterol, HDL cholesterol, non-HDL cholesterol, the ratio of LDL cholesterol and HDL-cholesterol, *cholinesterase* activity and HbA_1c_ decreased. After IG1, total cholesterol, LDL cholesterol and non-HDL cholesterol were lower compared to CG1. In IG2, MCV and the concentrations of uric acid in blood and urine increased compared to the baseline.

Previous studies have shown the influence of microalgae on the *cholinesterase* activity and their potential in Alzheimer’s therapy. *Scenedesmus obliquus* and *Dunaliella salina* have been shown to decrease and *Arthrospira platensis* has been shown to increase *cholinesterase* activity [63,64]. This is not yet fully understood. The decreased *cholinesterase* activity in IG1 was within the reference range for healthy subjects (89–215 µmol/L*s). The reduction in *cholinesterase* activity due to the *Chlorella* smoothie is probably not connected to health issues but most likely to the already-known influence of different microalgae fiber on *cholinesterase* activity.

Elevated HbA_1c_ has been associated with increased cardiovascular mortality [65,66]. In all participants of the NovAL study, HbA_1c_ was below 5.7%. The combination of menu plans according to the MoKaRi concept and the consumption of smoothies enriched with *Chlorella* seems to have a beneficial effect on blood glucose concentrations as HbA_1c_ was reduced in IG1. Fallah et al. identified the influence of various compounds from *Chlorella,* such as fibers, carotenoids and phytosterols, on fasting glucose concentrations, resulting in lower HbA_1c_ [67], yet no changes in HbA_1c_ of patients with diabetes mellitus type 2 were described after daily consumption of 1.5 mg of *Chlorella* over a period of eight weeks [68]. Most likely, the decrease in HbA_1c_ is a normal biological and methodological variation because there were no major effects on the HbA_1c_ expected due to the average lifespan of 120 day of erythrocytes and the short intervention period.

The consumption of the MoKaRi-based menu plans, rich in total fiber and low in SFA, caused reductions of cardiovascular risk factors such as total cholesterol and LDL cholesterol. Increased consumption of SFA correlates with higher concentrations of total cholesterol and LDL cholesterol [69]. Fibers can be prebiotics, stimulating the activity and growth of health-promoting bacteria in the colon [70]. Dietary fibers are also connected to decreased blood cholesterol by influencing cholesterol synthesis and therefore reducing cardiovascular risk [71]. This effect was enhanced by the additional daily intake of the *Chlorella* smoothie (10.4 g of fiber per smoothie) compared to the non-smoothie-receiving groups and the consumption of the *Microchloropsis* smoothie (8.6 g of fiber per smoothie). A meta-analysis investigated the effects of *Chlorella* supplementation on cardiovascular risk factors. Fibers, carotenoids, phytosterols and other bioactive compounds from *Chlorella* appear to have beneficial effects on reducing cholesterol, triglycerides, fasting glucose and blood pressure [67]. In the NovAL study, comparable changes in cholesterol concentrations were observed. By decreasing the absorption of cholesterol and influencing its metabolism, phytosterols from *Chlorella* have shown to maintain normal blood cholesterol concentrations in high-fat diets [72,73], yet we are unable to clarify which ingredient of *Chlorella* leads to the reduction in cholesterol concentrations in IG1. We assume that a combination of total fiber, phytosterols, carotenoids and other bioactive compounds is responsible.

We observed an increase in uric acid in blood and urine within both intervention groups that was not different from the control groups. Uric acid is a degradation product of the purine metabolism in humans. Increased concentrations of uric acid are linked with hypertension, atrial fibrillation, coronary artery disease, heart failure and chronic kidney disease [74,75,76]. However, it is still not clear whether serum uric acid is a marker for cardiovascular disease or a causal risk factor [74]. The smoothies used in the NovAL study contained in total 79 or 151 mg of purines per 350 mL smoothie, mostly from the microalgae. An increased purine intake causes higher uric acid serum concentrations and urinary excretion of uric acid [77,78]. We assume an association between purine intake by microalgae and the increase in uric acid in the intervention groups because of the metabolism of purines to uric acid in the human body. Furthermore, changes in blood viscosity by increased uric acid concentrations can cause higher concentrations of inter alia serum fibrinogen, which has been observed for IG1 [79]. Due to its involvement in blood clotting, elevated concentrations of fibrinogen are associated with coronary heart diseases and stroke and are therefore an important risk factor for cardiovascular diseases [80]. Elevated uric acid concentrations in the plasma after regular consumption of microalgae were detected in rats and male humans [81,82]. Previously, the authors declared their concerns for the consequences for human health of consuming up to 50 g of microalgae per day, yet the potential harm for developing gout and kidney stones due to the increased concentrations of purines or nucleic acids caused by regular consumption of microalgae could not be confirmed in a rat study [81,83]. The mean uric acid concentrations of the NovAL study participants fits with the reference range (143 to 339 µmol/L for women and 202 to 417 µmol/L for men) and were not different from the control groups. However, the increase of up to 15% uric acid in plasma and 43% in urine in both intervention groups might deteriorate health status, particularly in individuals with already slightly elevated, high-normal uric acid or impaired kidney function. This should, therefore, be considered in the evaluation of the long-term effects of regular microalgae consumption.

## 5. Conclusions

The consumption of 15 g/d of *Chlorella pyrenoidosa* for 14 days increased 25-hydroxyvitamin D_2_ serum concentrations, decreased selenium plasma concentrations and worsened iron status. Furthermore, cardiovascular risk factors such as total cholesterol, LDL cholesterol, the LDL-cholesterol to HDL-cholesterol ratio and non-HDL cholesterol were reduced. The *Microchloropsis salina*-enriched smoothies improved the fatty acid distribution in plasma lipids by increasing the LC n3 PUFA content and reducing n6/n3 PUFA ratio. The NovAL study is limited by its comparably low intake of microalgae per day and the short study period of 14 days. The study collective consisted of young and healthy participants, without known nutrient deficiencies or elevated cardiovascular risk factors. We assume that a longer consumption of higher dosages will result in larger effects on nutrient status, particularly in participants with nutrient deficiencies. The short study duration can only partially reflect the effect of *Chlorella pyrenoidosa* and *Microchloropsis salina* consumption on human health. Our findings indicate that *Chlorella pyrenoidosa* is a suitable vitamin D_2_ source and may have a positive effect on blood cholesterol. On the other hand, an elevated requirement of iron and selenium should be considered to prevent deficits of these nutrients. *Microchloropsis salina* is a suitable source of LC n3 PUFAs. Further investigations are needed to evaluate the influence of regular microalgae consumption on uric acid metabolism to avoid adverse effects.

## Figures and Tables

**Table 1:** Nutrient profile of both intervention products in total (350 mL smoothie per day).

Parameter	*Chlorella pyrenoidosa* Smoothie (IG1)	*Microchloropsis salina* Smoothie (IG2)
*Macronutrients*		
Fiber (g)	10.4	8.56
Fat (g)	3.69	4.77
Protein (g)	9.53	7.18
Carbohydrates (g)	26.6	29.4
*Vitamins*		
Vitamin B_12_ (µg)	<0.3	<0.3
Vitamin D_2_ (ng)	63,090	<1.05
Vitamin D_3_ (ng)	186	<0.15
α-Tocopherol (mg)	1.86	7.01
β-Tocopherol (mg)	n.d.	n.d.
γ-Tocopherol (mg)	<0.01	0.14
δ-Tocopherol (mg)	<12	<12
*Minerals and trace elements*		
Calcium (mg)	142	465
Magnesium (mg)	101	140
Iron (mg)	15.9	14.1
Iodine (µg)	33.8	6.62
Copper (µg)	232	495
Manganese (mg)	1.34	1.89
Nickel (µg)	5.10	180
Selenium (µg)	1.65	0.71
Zinc (µg)	660	900
*Purine*		
Adenine (mg)	26.5	65.0
Caffeine (mg)	<1	<1
Guanine (mg)	38.5	81.0
Uric acid (mg)	0.3	0.7
Hypoxanthine (mg)	7.9	0.8
Theobromine (mg)	<1	<1
6-Thioguanidine (mg)	<1	<1
Xanthine (mg)	2.5	0.5
Sum purine nitrogen (mg)	35.5	72.0

IG1, intervention group I; IG2 intervention group II; n.d., not detectable.

**Table 2:** Fatty acid profile of both intervention products (in mg/350 mL smoothie per day).

FAME	*Chlorella pyrenoidosa* Smoothie (IG1)	*Microchloropsis salina* Smoothie (IG2)
*SFA*		
C10:0	0.27	1.52
C12:0	0.60	13.31
C14:0	4.36	125.33
C15:0	1.24	6.72
C16:0	275.62	515.27
C17:0	3.26	7.64
C18:0	132.88	13.98
C20:0	4.28	1.85
C22:0	2.41	1.52
C24:0	5.93	0.56
*MUFA*		
C14:1n6	<0.1	2.16
C16:1n7	35.92	540.45
C17:1n7	1.48	<0.1
C18:1n9	717.32	290.00
C18:1n7	34.30	34.37
C20:1n9	2.24	0.91
C20:1n12	0.03	0.03
*n6 PUFA*		
C16:2n6	0.22	3.90
C18:2n6	390.23	89.47
C18:3n6	<0.1	7.83
C20:2n6	0.88	0.45
C20:3n6	<0.1	10.80
C20:4n6	<0.1	99.30
C22:4n6	<0.1	<0.1
C22:5n6	0.24	<0.1
*n3 PUFA*		
C18:3n3	35.32	29.98
C20:4n3	<0.1	1.45
C20:5n3	0.53	714.25
C22:5n3	<0.1	<0.1
C22:6n3	<0.1	<0.1
*Sum*		
SFA	430.85	687.70
MUFA	791.39	868.02
PUFA	428.12	957.83
n6-PUFA	391.97	211.95
n3-PUFA	36.15	745.88
n6/n3	10.84	0.28

FAME, fatty acid methyl esters; IG1, intervention group I; IG2 intervention group II; MUFA, monounsaturated fatty acids; n3, omega-3; n6, omega-6; n7, omega-7; n9, omega-9; n12, omega-12; PUFA, polyunsaturated fatty acids; SFA, saturated fatty acids.

**Table 3:** Nutrient status of the NovAL study participants based on blood and urine parameters (*n* = 72).

		Control Group I					Control Group II					Intervention Group I					Intervention Group II				
	Weeks	*n*	Md	/	IQR	*p*	*n*	Md	/	IQR	*p*	*n*	Md	/	IQR	*p*	*n*	Md	/	IQR	*p*
*Vitamins in blood*																					
Vitamin A	0	11	1.60	/	0.84	^a^	11	1.64	/	0.33	^a^	14	1.55	/	0.44	^a^	13	1.70	/	0.50	^a^
(µmol/L)	2	11	1.75	/	0.51	^a^	11	1.98	/	0.80	^a^	14	1.47	/	0.40	^a^	13	1.41	/	0.60	^a^
	Δ	11	−0.10	/	0.30	^a^	11	0.07	/	0.73	^a^	14	−0.17	/	0.34	^a^	13	−0.05	/	0.31	^a^
Vitamin B_1_	0	17	134	/	28	^a^	16	114	/	31	^a^	19	122	/	34	^a^	17	114	/	28	^a^
(nmol/L)	2	17	122	/	14	^a^	16	131	/	31	^a^	19	134	/	33	^a^	17	128	/	8	^a^
	Δ	17	−17	/	128	^a^	16	2.9	/	41.3	^a^	19	−2	/	65	^a^	17	1	/	44	^a^
Vitamin B_2_	0	19	211	/	33	^a^	17	209	/	45	^a^	19	195	/	33	^a^	17	205	/	31	^a^
(µg/L)	2	19	199	/	27	^a^	17	207	/	30	^a^	19	195	/	31	^a^	17	202	/	17	^a^
	Δ	19	−11	/	38	^a^	17	−9	/	35	^a^	19	1	/	39	^a^	17	−15	/	48	^a^
Vitamin B_6_	0	19	59.8	/	36.2	^a^	17	53.2	/	25.0	^a^	19	73.4	/	47.6	^a^	17	75.1	/	38.9	^a^
(nmol/L)	2	19	64.4	/	61.5	^a^	17	66.4	/	18.1	^a^	19	76.8	/	28.4	^a^	17	92.4	/	22.8	^a^
	Δ	19	5.6	/	34.0	^a^	17	10.4	/	19.3	^a^	19	2.2	/	16.9	^a^	17	10.5	/	22.5	^a^
Vitamin B_12_	0	19	228	/	196	^a^	17	292	/	142	^a^	19	244	/	67	^a^	17	295	/	137	^a^
(nmol/L)	2	19	242	/	202	^a^	17	271	/	162	^a^	19	291	/	70	^a^	17	283	/	144	^a^
	Δ	19	−5	/	25	^a^	17	−3	/	40	^a^	19	*24	/	24	^a^	17	0	/	46	^a^
Holo-trans-	0	19	60.8	/	16.3	^a^	17	68.8	/	42.4	^a^	19	69.8	/	19.5	^a^	17	63.2	/	27.7	^a^
cobalamin	2	19	58.8	/	27.7	^a^	17	64.3	/	24.2	^a^	19	79.2	/	36.6	^a^	17	66.7	/	40.7	^a^
(pmol/L)	Δ	19	2.6	/	14.2	^a^	17	−4.8	/	16.8	^a^	19	9.1	/	17.3	^a^	17	4.3	/	16.4	^a^
Vitamin C	0	19	5.3	/	2.5	^a^	17	4.4	/	4.1	^a^	19	5.7	/	2.7	^a^	17	5.5	/	2.2	^a^
(mg/L)	2	19	7.1	/	3.0	^a^	17	7.9	/	3.3	^a^	19	7.7	/	1.4	^a^	17	7.9	/	2.5	^a^
	Δ	19	1.8	/	2.7	^a^	17	* 2.6	/	2.5	^a^	19	* 1.9	/	4.2	^a^	17	* 3.3	/	3.4	^a^
25-Hydroxy-	0	18	3.40	/	1.82	^a^	17	2.50	/	0.77	^a^	18	2.83	/	1.32	^a^	16	2.52	/	1.60	^a^
vitamin D_2_	2	18	3.19	/	1.15	^a^	17	2.50	/	0.24	^a^	18	4.71	/	1.42	^b^	16	2.50	/	0.97	^a^
(nmol/L)	Δ	18	0.00	/	0.27	^a^	17	0.00	/	0.18	^a^	18	* 1.49	/	1.43	^b^	16	0.00	/	0.51	^a^
25-Hydroxy-	0	18	57.48	/	24.06	^a^	17	43.30	/	14.59	^a^	19	57.91	/	27.71	^a^	17	49.12	/	23.59	^a^
vitamin D_3_	2	18	47.29	/	20.21	^a^	17	37.27	/	9.15	^a^	19	48.38	/	19.97	^a^	17	39.55	/	39.42	^a^
(nmol/L)	Δ	18	* −6.81	/	9.59	^a^	17	* −3.97	/	5.09	^a^	19	* −4.31	/	7.82	^a^	17	* −7.44	/	6.67	^a^
Vitamin D_3_	0	17	2.04	/	11.78	^a^	17	1.52	/	1.17	^a^	18	4.13	/	6.95	^a^	16	2.36	/	6.10	^a^
(nmol/L)	2	17	1.63	/	3.25	^a^	17	1.32	/	0.79	^a^	18	2.87	/	3.35	^a^	16	1.64	/	2.87	^a^
	Δ	17	* −0.48	/	1.08	^a^	17	* −0.33	/	0.39	^a^	18	* −0.85	/	1.76	^a^	16	* −0.89	/	3.55	^a^
Vitamin E	0	8	30.5	/	5.4	^a^	9	29.5	/	10.6	^a^	9	30.7	/	9.6	^a^	9	29.4	/	8.2	^a^
(µmol/L)	2	8	30.7	/	5.0	^a^	9	30.7	/	5.1	^a^	9	27.6	/	6.3	^a^	9	30.7	/	7.7	^a^
	Δ	8	−3.7	/	31.0	^a^	9	−2.0	/	30.5	^a^	9	−6.0	/	21.1	^a^	9	1.0	/	12.7	^a^
Vitamin H	0	19	296	/	151	^a^	16	305	/	120	^a^	18	305	/	139	^a^	16	286	/	128	^a^
(ng/L)	2	19	277	/	124	^a^	16	284	/	138	^a^	18	374	/	170	^a^	16	345	/	158	^a^
	Δ	19	−21	/	203	^a^	16	−37	/	157	^a^	18	11	/	209	^a^	16	24	/	209	^a^
Folic acid	0	19	6.50	/	3.45	^a^	17	5.3	/	2.0	^a^	19	7.0	/	3.9	^a^	17	6.2	/	5.1	^a^
(µg/L)	2	19	6.90	/	4.35	^a^	17	7.2	/	2.1	^a^	19	7.2	/	4.6	^a^	17	6.7	/	3.3	^a^
	Δ	19	0.5	/	3.25	^a^	17	1.3	/	1.8	^a^	19	0.8	/	2.6	^a^	17	0.6	/	3.4	^a^
*Minerals and trace elements in blood*																					
Calcium	0	19	2.34	/	0.11	^a^	17	2.27	/	0.09	^a^	19	2.33	/	0.08	^a^	17	2.33	/	0.04	^a^
(mmol/L)	2	19	2.30	/	0.10	^a^	17	2.29	/	0.09	^a^	19	2.31	/	0.11	^a^	17	2.34	/	0.04	^a^
	Δ	19	−0.03	/	0.11	^a^	17	−0.01	/	0.09	^a^	19	−0.02	/	0.07	^a^	17	0.02	/	0.11	^a^
Potassium	0	19	3.75	/	0.26	^a^	17	4.02	/	0.21	^a^	19	3.89	/	0.38	^a^	17	3.91	/	0.47	^a^
(mmol/L)	2	19	3.81	/	0.21	^a^	17	3.99	/	0.30	^a^	19	3.90	/	0.43	^a^	17	3.95	/	0.40	^a^
	Δ	19	0.07	/	0.39	^a^	17	−0.08	/	0.25	^a^	19	−0.13	/	0.41	^a^	17	−0.01	/	0.30	^a^
Iodine	0	19	57.0	/	11.9	^a^	17	54.8	/	16.6	^a^	19	48.7	/	13.6	^a^	17	52.3	/	9.8	^a^
(µmol/L)	2	19	58.8	/	13.7	^a^	17	47.2	/	12.9	^a^	19	50.3	/	20.9	^a^	17	52.6	/	10.9	^a^
	Δ	19	−4.3	/	11.3	^a^	17	−7.8	/	11.5	^a^	19	−4.9	/	5.9	^a^	17	0.3	/	8.0	^a^
Selenium	0	19	1.44	/	0.27	^a^	17	1.28	/	0.55	^a^	19	1.48	/	0.36	^a^	17	1.38	/	0.50	^a^
(µmol/L)	2	19	1.43	/	0.29	^a^	17	1.24	/	0.38	^a^	19	1.35	/	0.33	^a^	17	1.30	/	0.24	^a^
	Δ	19	0.04	/	0.35	^a^	17	−0.13	/	0.57	^a^	19	* −0.28	/	0.40	^a^	17	−0.10	/	0.47	^a^
*Minerals and trace elements in urine*																					
Magnesium	0	19	1.69	/	0.98	^a^	17	2.05	/	0.82	^a^	19	1.90	/	0.96	^a^	17	2.05	/	0.44	^a^
(mmol/L)	2	19	1.91	/	0.90	^a^	17	1.92	/	0.81	^a^	19	1.77	/	2.24	^a^	17	2.22	/	0.65	^a^
	Δ	19	0.22	/	0.96	^a^	17	−0.10	/	0.78	^a^	19	0.15	/	0.60	^a^	17	−0.09	/	0.70	^a^
Sodium	0	19	64	/	24	^a^	17	56	/	36	^a^	19	66	/	46	^a^	17	51	/	37	^a^
(mmol/L)	2	19	60	/	29	^a^	17	51	/	36	^a^	19	55	/	27	^a^	17	56	/	28	^a^
	Δ	19	−8	/	32	^a^	17	−9	/	57	^a^	19	−1	/	44	^a^	17	6	/	15	^a^
Copper	0	19	0.29	/	0.05	^a^	17	0.29	/	0.00	^a^	19	0.29	/	0.05	^a^	17	0.29	/	0.02	^a^
(µmol/L)	2	19	0.29	/	0.00	^a^	17	0.29	/	0.02	^a^	19	0.29	/	0.10	^a^	17	0.31	/	0.08	^a^
	Δ	19	0.00	/	0.02	^a^	17	0.00	/	0.00	^a^	19	0.00	/	0.02	^a^	17	0.00	/	0.03	^a^
Selenium	0	19	0.19	/	0.19	^a^	17	0.14	/	0.08	^a^	19	0.16	/	0.12	^a^	17	0.16	/	0.05	^a^
(µmol/L)	2	19	0.14	/	0.10	^a^	17	0.10	/	0.10	^a^	19	0.10	/	0.10	^a^	17	0.14	/	0.11	^a^
	Δ	19	−0.06	/	0.10	^a^	17	−0.03	/	0.05	^a^	19	−0.08	/	0.20	^a^	17	−0.03	/	0.16	^a^
Zinc	0	19	4.1	/	3.1	^a^	17	3.5	/	2.9	^a^	19	3.5	/	3.2	^a^	17	2.8	/	1.2	^a^
(µmol/L)	2	19	3.7	/	2.1	^a^	17	3.1	/	2.6	^a^	19	2.8	/	3.2	^a^	17	3.4	/	2.3	^a^
	Δ	19	−0.3	/	3.2	^a^	17	−0.8	/	1.3	^a^	19	−0.7	/	1.7	^a^	17	0.8	/	2.2	^a^
*Iron status*																					
Iron	0	19	18.3	/	7.6	^a^	17	20.7	/	13.0	^a^	19	21.5	/	4.8	^a^	17	18.2	/	6.3	^a^
(µmol/L)	2	19	18.8	/	9.4	^a^	17	17.1	/	12.2	^a^	19	16.8	/	6.8	^a^	17	17.0	/	7.5	^a^
	Δ	19	−1.8	/	4.5	^a^	17	−1.0	/	8.0	^a^	19	* −6.4	/	8.4	^a^	17	−1.3	/	6.7	^a^
Transferrin	0	19	3.1	/	0.6	^a^	17	2.7	/	0.8	^a^	19	2.9	/	0.6	^a^	17	2.9	/	0.3	^a^
(g/L)	2	19	3.0	/	0.8	^a^	17	2.8	/	0.9	^a^	19	2.8	/	0.6	^a^	17	2.9	/	0.4	^a^
	Δ	19	0.0	/	0.3	^a^	17	0.1	/	0.3	^a^	19	−0.1	/	0.3	^a^	17	0.0	/	0.3	^a^
Transferrin	0	19	23.5	/	11.1	^a^	17	30.7	/	23.0	^a^	19	29.4	/	8.0	^a^	17	27.9	/	15.5	^a^
saturation	2	19	22.1	/	12.7	^a^	17	22.0	/	17.5	^a^	19	21.8	/	15.8	^a^	17	21.8	/	10.8	^a^
(%)	Δ	19	−3.0	/	4.1	^a^	17	−3.3	/	10.0	^a^	19	* −8.9	/	10.4	^a^	17	−1.9	/	7.1	^a^
Ferritin	0	19	35.7	/	62.3	^a^	17	74.3	/	83.2	^a^	19	38.0	/	32.0	^a^	17	34.7	/	128.2	^a^
(µg/L)	2	19	35.5	/	47.6	^a^	17	54.4	/	85.0	^a^	19	20.2	/	26.3	^a^	17	40.4	/	127.7	^a^
	Δ	19	−6.3	/	8.9	^a^	17	−7.5	/	15.2	^a^	19	* −13.9	/	14.8	^a^	17	−8.4	/	29.5	^a^
Hemoglobin	0	19	8.5	/	1.0	^a^	17	8.4	/	1.2	^a^	19	8.8	/	0.9	^a^	17	8.2	/	1.2	^a^
(mmol/L)	2	19	8.3	/	1.4	^a^	17	8.6	/	1.6	^a^	19	8.8	/	1.0	^a^	17	8.5	/	1.1	^a^
	Δ	19	0.0	/	0.4	^a^	17	0.1	/	0.4	^a^	19	−0.1	/	0.5	^a^	17	0.3	/	0.4	^a^

IQR, interquartile range; Md, median. Control group I = no defined background diet, control group II = defined background diet, intervention group I = defined background diet + *Chlorella pyrenoidosa* smoothie, intervention group II = defined background diet + *Microchloropsis salina* smoothie. Δ, difference between baseline and end of two-week period. ^ab^ Different superscript letters indicate significant differences between the groups (*p* < 0.05). * Significant differences between baseline and after the two-week study period within the study group (*p* < 0.05).

**Table 4:** Fatty acid profile in plasma lipids of the NovAL study participants (*n* = 72).

		Control Group I					Control Group II					Intervention Group I					Intervention Group II				
FAME	Weeks	*n*	Md	/	IQR	*p*	*n*	Md	/	IQR	*p*	*n*	Md	/	IQR	*p*	*n*	Md	/	IQR	*p*
*SFA (%)*																					
C14:0	0	17	0.93	/	0.35	^a^	16	0.92	/	0.38	^a^	19	0.83	/	0.36	^a^	17	0.88	/	0.36	^a^
	2	17	0.87	/	0.18	^a^	16	0.69	/	0.18	^b^	19	0.78	/	0.27	^a,b^	17	0.68	/	0.17	^b^
	Δ	17	0.16	/	0.44	^a^	16	−0.24	/	0.21	^a^	19	−0.16	/	0.29	^a^	17	−0.15	/	0.20	^a^
C15:0	0	17	0.27	/	0.07	^a^	16	0.28	/	0.08	^a^	19	0.27	/	0.05	^a^	17	0.25	/	0.08	^a^
	2	17	0.26	/	0.04	^a^	16	0.23	/	0.04	^a^	19	0.27	/	0.04	^a^	17	0.24	/	0.04	^a^
	Δ	17	0.01	/	0.04	^a^	16	−0.02	/	0.06	^a^	19	−0.02	/	0.02	^a^	17	−0.01	/	0.07	^a^
C16:0	0	17	21.17	/	2.75	^a^	16	20.87	/	2.23	^a^	19	20.90	/	1.71	^a^	17	20.23	/	1.07	^a^
	2	17	20.54	/	2.33	^a^	16	20.80	/	2.74	^a^	19	20.18	/	2.18	^a^	17	19.92	/	1.80	^a^
	Δ	17	−0.79	/	2.75	^a^	16	−1.09	/	1.68	^a^	19	−0.38	/	2.09	^a^	17	−0.06	/	1.77	^a^
C17:0	0	17	0.26	/	0.05	^a^	16	0.25	/	0.05	^a^	19	0.26	/	0.04	^a^	17	0.25	/	0.03	^a^
	2	17	0.25	/	0.02	^a^	16	0.27	/	0.05	^a^	19	0.29	/	0.03	^a^	17	0.31	/	0.04	^a^
	Δ	17	0.00	/	0.04	^a^	16	0.02	/	0.06	^a,b^	19	0.01	/	0.06	^a,b^	17	* 0.05	/	0.07	^b^
C18:0	0	17	6.59	/	1.27	^a^	16	6.89	/	0.64	^a^	19	6.72	/	0.86	^a^	17	6.98	/	0.69	^a^
	2	17	6.61	/	0.85	^a^	16	6.61	/	0.68	^a^	19	6.58	/	1.13	^a^	17	7.03	/	0.47	^a^
	Δ	17	0.10	/	1.01	^a^	16	0.17	/	0.94	^a^	19	0.01	/	0.79	^a^	17	−0.06	/	0.95	^a^
Sum	0	17	29.67	/	2.21	^a^	16	29.55	/	2.71	^a^	19	29.30	/	1.20	^a^	17	29.06	/	0.92	^a^
SFA	2	17	29.23	/	1.88	^a^	16	29.22	/	1.77	^a^	19	28.66	/	2.85	^a^	17	28.51	/	1.13	^a^
	Δ	17	0.04	/	3.34	^a^	16	−0.65	/	1.26	^a^	19	−0.28	/	2.24	^a^	17	−0.58	/	1.71	^a^
*MUFA (%)*																					
C16:1n7	0	17	1.71	/	0.69	^a^	16	1.55	/	0.63	^a^	19	1.77	/	0.62	^a^	17	1.46	/	0.61	^a^
	2	17	1.55	/	0.69	^a^	16	1.80	/	0.88	^a^	19	1.67	/	0.28	^a^	17	1.70	/	0.50	^a^
	Δ	17	−0.12	/	0.71	^a^	16	−0.05	/	0.44	^a^	19	0.00	/	0.62	^a^	17	−0.02	/	0.37	^a^
C18:1n9	0	17	20.34	/	2.61	^a^	16	20.21	/	4.53	^a^	19	21.16	/	1.81	^a^	17	20.55	/	3.52	^a^
	2	17	21.69	/	2.77	^a^	16	21.59	/	2.95	^a^	19	22.42	/	2.69	^a^	17	23.49	/	3.46	^a^
	Δ	17	0.35	/	1.53	^a^	16	2.10	/	2.71	^a^	19	* 1.27	/	1.98	^a^	17	1.23	/	2.19	^a^
C18:1n7	0	17	1.76	/	0.27	^a^	16	1.67	/	0.24	^a^	19	1.75	/	0.24	^a^	17	1.74	/	0.13	^a^
	2	17	1.75	/	0.25	^a^	16	1.89	/	0.26	^a^	19	1.89	/	0.36	^a^	17	2.05	/	0.29	^a^
	Δ	17	−0.03	/	0.24	^a^	16	0.26	/	0.35	^a^	19	* 0.18	/	0.23	^a^	17	* 0.25	/	0.19	^a^
Sum	0	17	24.72	/	2.69	^a^	16	24.20	/	3.54	^a^	19	25.57	/	3.20	^a^	17	24.05	/	3.79	^a^
MUFA	2	17	25.50	/	4.24	^a^	16	25.42	/	3.40	^a^	19	26.81	/	3.43	^a^	17	27.18	/	3.66	^a^
	Δ	17	0.84	/	2.53	^a^	16	2.27	/	2.78	^a^	19	1.29	/	2.14	^a^	17	1.69	/	2.56	^a^
*n6 PUFA (%)*																					
C18:2n6	0	17	31.35	/	5.06	^a^	16	30.70	/	2.09	^a^	19	29.53	/	5.03	^a^	17	30.03	/	3.77	^a^
	2	17	29.96	/	3.27	^a^	16	27.79	/	4.68	^a^	19	28.22	/	2.96	^a^	17	27.29	/	3.67	^a^
	Δ	17	−0.59	/	5.13	^a^	16	−2.12	/	3.82	^a^	19	−2.07	/	2.14	^a^	17	−2.23	/	4.47	^a^
C18:3n6	0	17	0.22	/	0.08	^a^	16	0.34	/	0.10	^a,b^	19	0.40	/	0.19	^b^	17	0.32	/	0.15	^a,b^
	2	17	0.22	/	0.11	^a^	16	0.25	/	0.12	^a^	19	0.29	/	0.16	^a^	17	0.27	/	0.17	^a^
	Δ	17	0.00	/	0.06	^a^	16	−0.06	/	0.13	^a^	19	−0.03	/	0.15	^a^	17	−0.04	/	0.07	^a^
C20:2n6	0	17	0.16	/	0.11	^a^	16	0.17	/	0.08	^a^	19	0.16	/	0.07	^a^	17	0.15	/	0.05	^a^
	2	17	0.19	/	0.05	^a^	16	0.19	/	0.06	^a^	19	0.19	/	0.05	^a^	17	0.16	/	0.04	^a^
	Δ	17	0.03	/	0.10	^a^	16	0.02	/	0.06	^a^	19	0.02	/	0.07	^a^	17	0.02	/	0.09	^a^
C20:3n6	0	17	1.36	/	0.44	^a^	16	1.77	/	0.55	^a^	19	1.69	/	0.42	^a^	17	1.47	/	0.32	^a^
	2	17	1.55	/	0.44	^a^	16	1.79	/	0.36	^a^	19	1.46	/	0.31	^a,b^	17	1.24	/	0.25	^b^
	Δ	17	0.13	/	0.12	^a^	16	−0.10	/	0.27	^a,b^	19	−0.10	/	0.37	^a,b^	17	−0.15	/	0.27	^b^
C20:4n6	0	17	6.40	/	1.67	^a^	16	6.75	/	1.58	^a^	19	6.95	/	1.64	^a^	17	6.76	/	1.32	^a^
	2	17	6.53	/	1.34	^a^	16	7.01	/	2.99	^a^	19	7.00	/	1.22	^a^	17	6.91	/	1.35	^a^
	Δ	17	0.22	/	0.96	^a^	16	0.61	/	2.23	^a^	19	−0.02	/	1.56	^a^	17	0.20	/	0.88	^a^
C22:4n6	0	17	0.16	/	0.05	^a^	16	0.18	/	0.04	^a^	19	0.17	/	0.02	^a^	17	0.18	/	0.03	^a^
	2	17	0.17	/	0.05	^a^	16	0.19	/	0.03	^a^	19	0.18	/	0.03	^a^	17	0.16	/	0.02	^a^
	Δ	17	0.02	/	0.03	^a^	16	0.00	/	0.04	^a^	19	0.02	/	0.04	^a^	17	−0.01	/	0.01	^a^
C22:5n6	0	17	0.12	/	0.05	^a^	16	0.13	/	0.05	^a^	19	0.13	/	0.04	^a^	17	0.12	/	0.04	^a^
	2	17	0.12	/	0.06	^a^	16	0.16	/	0.05	^a^	19	0.13	/	0.04	^a^	17	0.11	/	0.04	^a^
	Δ	17	0.00	/	0.04	^a^	16	0.02	/	0.03	^a^	19	0.01	/	0.05	^a^	17	−0.01	/	0.02	^a^
Sum n6	0	17	40.16	/	3.60	^a^	16	40.31	/	1.28	^a^	19	38.49	/	5.93	^a^	17	39.86	/	2.71	^a^
PUFA	2	17	38.99	/	2.86	^a^	16	38.90	/	2.40	^a^	19	38.08	/	4.22	^a^	17	36.69	/	3.31	^a^
	Δ	17	−0.52	/	5.60	^a^	16	−1.98	/	3.30	^a^	19	−1.58	/	1.96	^a^	17	−1.99	/	4.32	^a^
*n3 PUFA (%)*																					
C18:3n3	0	17	0.55	/	0.49	^a^	16	0.43	/	0.20	^a^	19	0.50	/	0.20	^a^	17	0.57	/	0.19	^a^
	2	17	0.47	/	0.19	^a^	16	0.54	/	0.24	^a^	19	0.59	/	0.29	^a^	17	0.62	/	0.25	^a^
	Δ	17	−0.03	/	0.32	^a^	16	0.10	/	0.23	^a^	19	0.02	/	0.17	^a^	17	0.03	/	0.43	^a^
C20:5n3	0	17	0.37	/	0.25	^a^	16	0.45	/	0.28	^a^	19	0.49	/	0.21	^a^	17	0.59	/	0.35	^a^
	2	17	0.40	/	0.21	^a^	16	0.37	/	0.21	^a^	19	0.41	/	0.25	^a^	17	0.88	/	0.51	^b^
	Δ	17	−0.05	/	0.25	^a^	16	−0.09	/	0.31	^a^	19	−0.10	/	0.18	^a^	17	* 0.24	/	0.65	^b^
C22:5n3	0	17	0.32	/	0.15	^a^	16	0.36	/	0.14	^a^	19	0.37	/	0.16	^a^	17	0.46	/	0.14	^a^
	2	17	0.36	/	0.18	^a^	16	0.40	/	0.12	^a^	19	0.43	/	0.21	^a^	17	0.65	/	0.13	^b^
	Δ	17	0.05	/	0.11	^a^	16	0.03	/	0.10	^a^	19	0.01	/	0.08	^a^	17	* 0.17	/	0.10	^b^
C22:6n3	0	17	1.51	/	0.37	^a^	16	1.45	/	0.47	^a^	19	1.49	/	0.63	^a^	17	1.70	/	0.83	^a^
	2	17	1.41	/	0.64	^a^	16	1.63	/	0.58	^a^	19	1.43	/	0.38	^a^	17	1.52	/	0.44	^a^
	Δ	17	0.09	/	0.43	^a^	16	0.18	/	0.43	^a^	19	−0.06	/	0.28	^a^	17	−0.24	/	0.54	^a^
Sum n3	0	17	2.83	/	0.78	^a^	16	2.66	/	0.91	^a^	19	3.05	/	0.96	^a^	17	3.54	/	1.21	^a^
PUFA	2	17	2.72	/	0.80	^a,b^	16	3.03	/	0.69	^a,b^	19	2.95	/	0.94	^a^	17	3.86	/	0.85	^b^
	Δ	17	−0.01	/	0.93	^a^	16	0.10	/	0.70	^a^	19	−0.09	/	0.67	^a^	17	0.48	/	1.13	^a^
Sum	0	17	2.04	/	0.80	^a^	16	2.20	/	0.76	^a^	19	2.50	/	0.90	^a^	17	2.92	/	1.04	^a^
LC n3	2	17	2.00	/	1.04	^a,b^	16	2.44	/	0.51	^a,b^	19	2.28	/	0.87	^a^	17	3.08	/	1.03	^b^
PUFA	Δ	17	0.11	/	0.64	^a^	16	0.02	/	0.60	^a^	19	−0.10	/	0.42	^a^	17	0.17	/	1.24	^a^
Sum	0	17	42.80	/	5.50	^a^	16	43.08	/	1.31	^a^	19	41.95	/	6.11	^a^	17	43.71	/	2.66	^a^
PUFA	2	17	41.82	/	3.46	^a^	16	41.94	/	2.57	^a^	19	40.57	/	3.05	^a^	17	40.61	/	4.63	^a^
	Δ	17	−0.57	/	5.98	^a^	16	−1.72	/	2.49	^a^	19	−1.45	/	2.73	^a^	17	−1.45	/	4.34	^a^
n6/n3	0	17	14.12	/	3.77	^a^	16	14.93	/	5.29	^a^	19	13.00	/	3.30	^a^	17	11.44	/	2.80	^a^
ratio	2	17	14.14	/	4.59	^a^	16	12.92	/	2.50	^a,b^	19	13.70	/	4.85	^a^	17	9.97	/	2.03	^b^
	Δ	17	−0.11	/	3.49	^a^	16	−2.19	/	4.10	^a^	19	−0.60	/	3.37	^a^	17	−1.44	/	2.59	^a^

FAME, fatty acid methyl esters; IQR, interquartile range; LC, long chain; Md, median; MUFA, monounsaturated fatty acids; n3, omega-3; n6, omega-6; n7, omega-7; n9, omega-9; PUFA, polyunsaturated fatty acids; SFA, saturated fatty acids. Control group I = no defined background diet, control group II = defined background diet, intervention group I = defined background diet + *Chlorella pyrenoidosa* smoothie, intervention group II = defined background diet + *Microchloropsis salina* smoothie. Δ, difference between baseline and end of the two-week period. ^ab^ Different superscript letters indicate significant differences between the groups (*p* < 0.05). * Significant differences between baseline and after the two-week study period within the study group (*p* < 0.05).

**Table 5:** Safety parameters of the NovAL study participants (*n* = 72).

		Control Group I					Control Group II					Intervention Group I					Intervention Group II				
	Weeks	*n*	Md	/	IQR	*p*	*n*	Md	/	IQR	*p*	*n*	Md	/	IQR	*p*	*n*	Md	/	IQR	*p*
*Liver and kidney function*																					
Alanine ami-	0	19	0.25	/	0.15	^a^	17	0.28	/	0.16	^a^	19	0.34	/	0.21	^a^	17	0.26	/	0.23	^a^
notransferase	2	19	0.28	/	0.18	^a^	17	0.34	/	0.20	^a^	19	0.34	/	0.16	^a^	17	0.27	/	0.12	^a^
(µmol/L*s)	Δ	19	0.02	/	0.07	^a^	17	0.01	/	0.09	^a^	19	0.00	/	0.11	^a^	17	−0.03	/	0.04	^a^
Aspartate	0	19	0.33	/	0.09	^a^	17	0.31	/	0.11	^a^	19	0.36	/	0.11	^a^	17	0.35	/	0.16	^a^
aminotrans-	2	19	0.33	/	0.08	^a^	17	0.36	/	0.12	^a^	19	0.36	/	0.13	^a^	17	0.36	/	0.13	^a^
ferase (s)	Δ	19	0.01	/	0.08	^a^	17	−0.01	/	0.09	^a^	19	0.00	/	0.06	^a^	17	0.00	/	0.06	^a^
Cholin-	0	19	114	/	39	^a^	17	114	/	18	^a^	19	120	/	19	^a^	17	117	/	32	^a^
esterase	2	19	110	/	34	^a^	17	116	/	22	^a^	19	111	/	18	^a^	17	115	/	31	^a^
(µmol/L*s)	Δ	19	−4	/	10	^a,b^	17	1	/	8	^a^	19	* −6	/	6	^b^	17	1	/	16	^a,b^
γ-glutamyl-	0	19	0.22	/	0.07	^a^	17	0.20	/	0.05	^a^	19	0.19	/	0.07	^a^	17	0.19	/	0.10	^a^
transferase	2	19	0.21	/	0.08	^a^	17	0.19	/	0.04	^a^	19	0.18	/	0.06	^a^	17	0.17	/	0.09	^a^
(µmol/L*s)	Δ	19	0.00	/	0.02	^a^	17	0.00	/	0.02	^a^	19	−0.01	/	0.02	^a^	17	−0.02	/	0.04	^a^
Lactate de-	0	19	2.50	/	0.44	^a^	17	2.53	/	0.51	^a^	19	2.68	/	0.34	^a^	17	2.56	/	0.40	^a^
hydrogenase	2	19	2.64	/	0.27	^a^	17	2.68	/	0.32	^a^	19	2.64	/	0.40	^a^	17	2.74	/	0.37	^a^
(µmol/L*s)	Δ	19	0.15	/	0.12	^a^	17	0.17	/	0.20	^a^	19	0.10	/	0.40	^a^	17	0.14	/	0.15	^a^
*Clotting*																					
Activated par-	0	19	29.0	/	2.7	^a^	17	29.5	/	3.0	^a^	19	29.7	/	2.9	^a^	17	30.5	/	3.0	^a^
tial thrombo-	2	19	28.4	/	3.0	^a^	17	30.0	/	3.9	^a^	19	30.0	/	2.6	^a^	17	30.1	/	3.9	^a^
plastin time (s)	Δ	19	−0.3	/	1.0	^a^	17	0.3	/	1.3	^a^	19	0.3	/	1.1	^a^	17	0.3	/	1.8	^a^
Fibrinogen	0	19	2.3	/	0.4	^a^	17	2.3	/	0.8	^a^	19	2.4	/	0.5	^a^	17	2.1	/	0.4	^a^
(nmol/L)	2	19	2.4	/	0.3	^a^	17	2.4	/	0.7	^a^	19	2.7	/	0.7	^a^	17	2.3	/	0.6	^a^
	Δ	19	0.1	/	0.3	^a^	17	0.1	/	0.4	^a^	19	* 0.2	/	0.5	^a^	17	0.2	/	0.4	^a^
Quick	0	19	90	/	14	^a^	17	92	/	18	^a^	19	90	/	10	^a^	17	88	/	8	^a^
(%)	2	19	93	/	13	^a^	17	87	/	13	^a^	19	89	/	16	^a^	17	91	/	7	^a^
	Δ	19	2	/	8	^a^	17	−1	/	9	^a^	19	−1	/	10	^a^	17	1	/	7	^a^
International	0	19	1.1	/	0.1	^a^	17	1.1	/	0.2	^a^	19	1.1	/	0.1	^a^	17	1.1	/	0.0	^a^
normalized	2	19	1.1	/	0.1	^a^	17	1.1	/	0.1	^a^	19	1.1	/	0.1	^a^	17	1.1	/	0.1	^a^
ratio	Δ	19	0.0	/	0.1	^a^	17	0.0	/	0.1	^a^	19	0.0	/	0.0	^a^	17	0.0	/	0.0	^a^
*Blood count*																					
Hematocrit	0	19	0.41	/	0.05	^a^	17	0.40	/	0.07	^a^	19	0.42	/	0.03	^a^	17	0.40	/	0.04	^a^
(%)	2	19	0.40	/	0.06	^a^	17	0.41	/	0.07	^a^	19	0.41	/	0.04	^a^	17	0.41	/	0.03	^a^
	Δ	19	0.00	/	0.03	^a^	17	0.00	/	0.02	^a^	19	0.00	/	0.02	^a^	17	0.01	/	0.02	^a^
MCH	0	19	1.81	/	0.15	^a^	17	1.84	/	0.12	^a^	19	1.85	/	0.10	^a^	17	1.85	/	0.10	^a^
(fmol)	2	19	1.84	/	0.13	^a^	17	1.87	/	0.12	^a^	19	1.88	/	0.13	^a^	17	1.86	/	0.12	^a^
	Δ	19	0.01	/	0.03	^a^	17	0.01	/	0.03	^a^	19	0.00	/	0.05	^a^	17	0.01	/	0.04	^a^
MCHC	0	19	20.7	/	0.5	^a^	17	20.9	/	0.6	^a^	19	21.0	/	0.9	^a^	17	20.8	/	0.7	^a^
(mmol/L)	2	19	20.7	/	0.55	^a^	17	20.9	/	0.5	^a^	19	21.0	/	0.7	^a^	17	20.8	/	0.7	^a^
	Δ	19	0.0	/	0.3	^a^	17	0.0	/	0.5	^a^	19	−0.1	/	0.3	^a^	17	−0.2	/	0.3	^a^
MCV	0	19	88	/	5	^a^	17	89	/	4	^a^	19	89	/	3	^a^	17	87	/	4	^a^
(fl)	2	19	89	/	5	^a^	17	89	/	4	^a^	19	90	/	5	^a^	17	89	/	3	^a^
	Δ	19	0	/	1	^a^	17	0	/	1	^a^	19	* 1	/	2	^a^	17	* 1	/	1	^b^
Red cell	0	19	12.2	/	0.7	^a^	17	12.4	/	0.3	^a^	19	12.3	/	0.7	^a^	17	12.4	/	1.0	^a^
distribution	2	19	12.2	/	0.8	^a^	17	12.4	/	0.7	^a^	19	12.3	/	0.5	^a^	17	12.3	/	1.1	^a^
width (%)	Δ	19	−0.1	/	0.4	^a^	17	0.0	/	0.1	^a^	19	0.0	/	0.2	^a^	17	0.0	/	0.2	^a^
*Parameters in urine*																					
Albumin	0	19	4	/	6	^a^	17	3	/	1	^a^	19	3	/	0	^a^	17	3	/	0	^a^
(mg/L)	2	19	3	/	1	^a^	17	3	/	0	^a^	19	3	/	0	^a^	17	3	/	2	^a^
	Δ	19	−2	/	7	^a^	17	0	/	0	^a^	19	0	/	1	^a^	17	0	/	0	^a^
Creatinine	0	19	5.3	/	4.0	^a^	17	6.0	/	3.6	^a^	19	5.1	/	2.9	^a^	17	4.7	/	1.5	^a^
(mmol/L)	2	19	4.7	/	3.7	^a^	17	4.3	/	2.3	^a^	19	4.7	/	6.5	^a^	17	5.7	/	3.4	^a^
	Δ	19	−0.3	/	3.1	^a^	17	−0.5	/	2.2	^a^	19	−0.4	/	2.3	^a^	17	0.1	/	2.4	^a^

IQR, interquartile range; MCH, mean corpuscular hemoglobin; MCHC, mean corpuscular hemoglobin concentration; MCV, mean corpuscular volume; Md, median. Control group I = no defined background diet, control group II = defined background diet, intervention group I = defined background diet + *Chlorella pyrenoidosa* smoothie, intervention group II = defined background diet + *Microchloropsis salina* smoothie. Δ, difference between baseline and end of the 2-week period. ^ab^ Different superscript letters indicate significant differences between the groups (*p* < 0.05). * Significant differences between baseline and after the two-week study period within the study group (*p* < 0.05).

**Table 6:** Risk factors for cardiovascular diseases and diabetes mellitus type 2 of the NovAL study participants (*n* = 72).

		Control Group I					Control Group II					Intervention Group I					Intervention Group II				
	Weeks	*n*	Md	/	IQR	*p*	*n*	Md	/	IQR	*p*	*n*	Md	/	IQR	*p*	*n*	Md	/	IQR	*p*
*Cardiovascular factors*																					
Cholesterol	1	19	4.16	/	0.80	^a^	17	4.20	/	1.21	^a^	19	4.44	/	0.72	^a^	17	4.06	/	1.12	^a^
(mmol/L)	2	19	4.25	/	0.45	^a^	17	3.69	/	1.10	^a,b^	19	3.45	/	0.70	^b^	17	3.85	/	1.06	^a,b^
	Δ	19	0.0	/	0.52	^a^	17	−0.39	/	0.53	^a^	19	* −0.82	/	0.55	^b^	17	−0.25	/	0.83	^a^
HDL	1	19	1.71	/	0.72	^a^	17	1.76	/	0.89	^a^	19	1.71	/	0.59	^a^	17	1.70	/	0.26	^a^
cholesterol	2	19	1.47	/	0.54	^a^	17	1.79	/	0.84	^a^	19	1.44	/	0.47	^a^	17	1.52	/	0.57	^a^
(mmol/L)	Δ	19	−0.11	/	0.23	^a^	17	−0.07	/	0.28	^a^	19	* −0.17	/	0.25	^a^	17	−0.11	/	0.29	^a^
LDL	1	19	2.62	/	0.62	^a^	17	2.20	/	0.58	^a^	19	2.45	/	0.72	^a^	17	2.26	/	0.84	^a^
cholesterol	2	19	2.45	/	0.53	^a^	17	1.94	/	0.39	^b^	19	1.84	/	0.57	^b^	17	2.07	/	0.49	^b^
(mmol/L)	Δ	19	0.02	/	0.34	^a^	17	−0.48	/	0.66	^a,b^	19	* −0.71	/	0.40	^b^	17	−0.27	/	0.76	^a,b^
LDL choles-	1	19	1.48	/	0.95	^a^	17	1.12	/	1.03	^a^	19	1.45	/	0.84	^a^	17	1.36	/	0.62	^a^
terol/HDL	2	19	1.62	/	1.05	^a^	17	1.07	/	0.69	^b^	19	1.25	/	0.61	^a,b^	17	1.34	/	0.60	^a,b^
cholesterol	Δ	19	0.07	/	0.23	^a^	17	−0.14	/	0.41	^a,b^	19	* −0.23	/	0.16	^b^	17	−0.14	/	0.25	^a,b^
non-HDL	1	19	2.55	/	0.74	^a^	17	2.27	/	0.63	^a^	19	2.62	/	1.02	^a^	17	2.36	/	0.91	^a^
cholesterol	2	19	2.80	/	0.78	^a^	17	2.03	/	0.70	^a,b^	19	2.03	/	0.58	^b^	17	2.31	/	0.52	^a,b^
(mmol/L)	Δ	19	0.05	/	0.35	^a^	17	−0.37	/	0.63	^a,b^	19	* −0.61	/	0.44	^b^	17	−0.07	/	0.25	^a,b^
MDA LDL	1	19	47.7	/	33.1	^a^	17	44.3	/	34.4	^a^	19	47.8	/	16.6	^a^	17	50.4	/	32.6	^a^
cholesterol	2	19	53.4	/	30.0	^a^	17	41.2	/	29.6	^a^	19	44.8	/	28.2	^a^	17	56.1	/	35.7	^a^
(U/L)	Δ	19	6.4	/	27.7	^a^	17	8.8	/	31.6	^a^	19	−6.9	/	24.8	^a^	17	−3.5	/	42.8	^a^
Triglyceride	1	19	0.69	/	0.24	^a^	17	0.63	/	0.44	^a^	19	0.72	/	0.49	^a^	17	0.68	/	0.26	^a^
(mmol/L)	2	19	0.74	/	0.69	^a^	17	0.69	/	0.37	^a^	19	0.64	/	0.29	^a^	17	0.71	/	0.39	^a^
	Δ	19	0.00	/	0.21	^a^	17	0.06	/	0.17	^a^	19	−0.03	/	0.19	^a^	17	0.01	/	0.19	^a^
Uric acid	1	19	243	/	73	^a^	17	229	/	74	^a^	19	231	/	87	^a^	17	264	/	107	^a^
(µmol/L)	2	19	258	/	65	^a^	17	243	/	47	^a^	19	253	/	84	^a^	17	294	/	108	^a^
	Δ	19	−5	/	47	^a^	17	20	/	39	^a^	19	* 29	/	46	^a^	17	* 18	/	48	^a^
Uric acid in	1	19	1424	/	1119	^a^	17	1459	/	781	^a^	19	1229	/	829	^a^	17	1058	/	502	^a^
urine	2	19	1423	/	613	^a^	17	1322	/	377	^a^	19	1286	/	852	^a^	17	1451	/	1449	^a^
(µmol/L)	Δ	19	−1	/	748	^a^	17	34	/	818	^a^	19	* 48	/	424	^a^	17	* 361	/	845	^a^
*Diabetes factors*																					
Glucose	1	19	4.9	/	0.4	^a^	17	5.0	/	0.4	^a^	19	4.9	/	0.5	^a^	17	5.1	/	0.3	^a^
(mmol/L)	2	19	5.1	/	0.5	^a^	17	5.1	/	0.6	^a^	19	5.0	/	0.6	^a^	17	5.0	/	0.8	^a^
	Δ	19	0.1	/	0.3	^a^	17	0.2	/	0.6	^a^	19	−0.2	/	0.6	^a^	17	0.1	/	0.4	^a^
Insulin	1	19	6.1	/	4.9	^a^	17	6.6	/	6.5	^a^	19	7.0	/	4.2	^a^	17	6.1	/	5.5	^a^
(mU/L)	2	19	6.8	/	4.4	^a^	17	6.3	/	4.1	^a^	19	7.3	/	4.3	^a^	17	6.5	/	3.0	^a^
	Δ	19	0.7	/	2.4	^a^	17	0.6	/	2.8	^a^	19	0.7	/	2.9	^a^	17	0.4	/	2.3	^a^
Hemoglobin	1	19	5.10	/	0.20	^a^	17	5.00	/	0.30	^a^	19	5.10	/	0.35	^a^	17	5.00	/	0.40	^a^
A1c (%)	2	19	5.00	/	0.30	^a^	17	5.00	/	0.20	^a^	19	5.00	/	0.60	^a^	17	5.00	/	0.30	^a^
	Δ	19	−0.10	/	0.15	^a^	17	0.00	/	0.20	^a^	19	* −0.10	/	0.20	^a^	17	0.00	/	0.40	^a^

HDL, high density lipoprotein; IQR, interquartile range; LDL, low-density lipoprotein; Md, median; MDA, malondialdehyde-modified. Control group I = no defined background diet, control group II = defined background diet, intervention group I = defined background diet + *Chlorella pyrenoidosa* smoothie, intervention group II = defined background diet + *Microchloropsis salina* smoothie. Δ, difference between baseline and end of the 2-week period. ^ab^ Different superscript letters indicate significant differences between the groups (*p* < 0.05). * Significant differences between baseline and after the two-week study period within the study group (*p* < 0.05).

## Data Availability

The data presented in this study are available on request from the corresponding author.

## Supplementary Materials

The following supporting information can be downloaded at https://www.mdpi.com/article/10.3390/nu15071645/s1. Table S1: Analyzed nutrients with the used method/instruments and performing institute from both intervention products; Table S2: Analyzed parameters with the used method/instruments, reference range and performing institute; Table S3: Lifestyle and socioeconomic status—NovAL study (*n* = 72); Table S4: Energy and nutrient intake of the NovAL study participants in the week before starting the intervention (self-reports, 5 days, *n* = 72); Table S5: Anthropometric data composition and blood pressure of the NovAL study participants at the last blood drawing (*n* = 72), body; Table S6: Further blood parameters of the NovAL study participants (*n* = 72).
