# Supplementary material for: Impact of Regular Intake of Microalgae on Nutrient Supply and Cardiovascular Risk Factors: Results from the NovAL Intervention Study
Source: Nutrients. 2023 Mar 28;15(7):1645. doi: 10.3390/nu15071645 (PMC10097350; doi:10.3390/nu15071645)
Supplement: Supplementary file 1 [file nutrients-15-01645-s001.zip › nutrients-2287164-supplementary.pdf]

Table S1. Analyzed nutrients with the used method/instruments and performing institute from both intervention products

Parameter	Method/instrument	Institution
**Fiber** (g)	BIOQUANT Total Dietary Fiber kit (Merck, Darmstadt, Germany)	FSU
**Fat** (g)	SOXTHERM (C. Gerhardt, Königswinter, Germany)	FSU
**Protein** (g)	DIN EN ISO 14891 2002-07	FSU
**Carbohydrates** (g)	calculated	FSU
**Vitamin B_{12}** (µg)	HPLC-MS/MS (Shimadzu, Duisburg, Germany)	TeLA GmbH
**Vitamin D** (ng)	LC-MS/MS (Sciex, Darmstadt, Germany)	MLU
**Tocopherols** (mg)	HPLC (Agilent Technologies, Santa Clara, California, USA)	MLU
**Minerals and trace elements** (µg - mg)	LC-MS/MS (Chromsystems Instruments & Chemicals, Germany)	SGS Analytics Germany GmbH
**Purine** (mg)	HPLC-MS/MS (Shimadzu, Duisburg, Germany)	Jena Bios GmbH
**Fatty acids** (mg)	GC-FID (Shimadzu, Duisburg, Germany)	FSU

FSU, Junior Research Group Nutritional Concepts, Institute of Nutritional Sciences, Friedrich Schiller University Jena; GC-FID, gas chromatography-flame ionization detector; HPLC, High-performance liquid chromatography; HPLC-MS/MS, High-performance liquid chromatography- mass spectrometry/ mass spectrometry; LC-MS/MS, Liquid chromatography–mass spectrometry/ mass spectrometry; MLU, Institute of Agricultural and Nutritional Science, Martin Luther University Halle-Wittenberg; UP, Department of Food Chemistry, Institute of Nutritional Science, University of Potsdam

Table S2. Analyzed parameters with the used method/instruments, reference range and performing institute

Parameter	Method/instrument	Reference range	Institution/reference
Plasma, serum			
**Total cholesterol** (mmol/l)	Abbott Architect CI 16200 analyzer (Abbott, Wiesbaden, Germany)	< 5.2	ICCL‡
**LDL-cholesterol** (mmol/l)	Abbott Architect CI 16200 analyzer (Abbott, Wiesbaden, Germany)	< 3.35	ICCL‡
**HDL-cholesterol** (mmol/l)	Abbott Architect CI 16200 analyzer (Abbott, Wiesbaden, Germany)	> 1.03	ICCL‡
**MDA-LDL** (U/l)	ELISA (ImmBioMed GmbH, Germany)	N/A	Dianovis GmbH
**Triglycerides** (mmol/l)	Abbott Architect CI 16200 analyzer (Abbott, Wiesbaden, Germany)	< 1.7	ICCL‡
**High-sensitive C-reactive protein** (mg/dl)	Abbott Architect CI 16200 analyzer (Abbott, Wiesbaden, Germany)	< 0.3	ICCL‡
**Homocysteine** (µmol/l)	HPLC (Shimadzu, Duisburg, Germany)	5 ≤ 15 Weikert et al. 2020	ICCL‡

**Methyl malonic acid** (µg/l)	LC-MS/MS (Chromsystems Instruments & Chemicals, Germany)	8.6 ≤ 32.0	Dianovis GmbH
**Iron** (µmol/l)	Abbott Architect CI 16200 analyzer (Abbott, Wiesbaden, Germany)	Male: 4.1 - 30.1<br>Female: 4.1 - 26.7	ICCL[‡]
**Ferritin** (µg/l)	Abbott Architect CI 16200 analyzer (Abbott, Wiesbaden, Germany)	Male: 30 - 400<br>Female: 13 - 150	ICCL[‡]
**Transferrin** (g/l)	Abbott Architect CI 16200 analyzer (Abbott, Wiesbaden, Germany)	2.0 - 3.6	ICCL[‡]
**Hemoglobin** (mmol/l)	XN 1000 (Sysmex, Norderstedt, Germany)	7.6 - 9.5	ICCL[‡]
**MCV** (fl)	XN 1000 (Sysmex, Norderstedt, Germany)	80 - 96	ICCL[‡]
**Vitamin A** (µmol/l)	HPLC (Shimadzu, Japan)	1.46 - 2.85 Weikert et al. 2020	ICCL[‡]
**Vitamin B_1** (nmol/l)	HPLC (Shimadzu, Japan)	47 - 1412	ICCL[‡]/ Chromsystems & Chemicals, DIN EN ISO 9001:2008 & DIN EN ISO 13485:2007-10
**Vitamin B_2** (µg/l)	HPLC (RECIPE Chemicals + Instruments GmbH, Germany)	180 - 295	Dianovis GmbH
**Vitamin B_6** (nmol/l)	HPLC (Shimadzu, Japan)	14.6 - 72.8	ICCL[‡]/ Chromsystems & Chemicals, DIN EN ISO 9001:2008 & DIN EN ISO 13485:2007-10
**Holotranscobalamin** (pmol/l)	Abbott Architect CI 16200 analyzer (Abbott, Wiesbaden, Germany)	> 37.52	ICCL[‡]
**Vitamin C** (mg/l)	HPLC (RECIPE Chemicals + Instruments GmbH, Germany)	4 - 15	Dianovis GmbH
**25-Hydroxyvitamin D_2** (nmol/l)	LC coupled to QTRAP 5500 MS-system using the MassChrome kit	No information available	Institute of Agricultural and Nutritional Science, Martin Luther University Halle-Wittenberg, Germany
**25-Hydroxyvitamin D_3** (nmol/l)	*Identical to 25-Hydroxyvitamin D_2*	> 75	*Identical to 25-Hydroxyvitamin D_2*
**Vitamin D_2** (nmol/l)	*Identical to 25-Hydroxyvitamin D_2*	No information available	*Identical to 25-Hydroxyvitamin D_2*
**Vitamin D_3** (nmol/l)	*Identical to 25-Hydroxyvitamin D_2*	No information available	*Identical to 25-Hydroxyvitamin D_2*
**α-Tocopherol** (µmol/l)	HPLC (Shimadzu, Japan)	11.6 - 46.4	ICCL[‡]
**Biotin** (ng/l)	IDK Biotin ELISA (Immundiagnostik AG, Bensheim, Germany)	> 250	Dianovis GmbH
**Folate** (µg/l)	Abbott Architect CI 16200 analyzer (Abbott, Wiesbaden, Germany)	3.9 - 26.8	ICCL[‡]
**Calcium** (mmol/l)	Abbott Architect CI 16200 analyzer (Abbott, Wiesbaden, Germany)	2.15 - 2.50	ICCL[‡]
**Potassium** (mmol/l)	Abbott Architect CI 16200 analyzer (Abbott, Wiesbaden, Germany)	3.4 - 4.5	ICCL[‡]
24 h urine			

**Magnesium** (mmol/24h)	Abbott Architect CI 16200 analyzer (Abbott, Wiesbaden, Germany)	No information available	ICCL‡
**Sodium** (mmol/24h)	Abbott Architect CI 16200 analyzer (Abbott, Wiesbaden, Germany)	94 - 222	ICCL‡
**Selenium** (µmol/ 24h)	AAS ZEEnit 60 (Analytik Jena, Jena, Germany)	No information available	ICCL‡
**Zinc** (µmol/24h)	AAS 5 FL (Analytik Jena, Germany)	No information available	ICCL‡
**Iodine** (µl/l)	PerkinElmer NexION 300D ICP-MS (PerkinElmer, Waltham, Massachusetts, USA)	100 - 200	Dianovis GmbH
**Albumin** (mg/l)	Abbott Architect CI 16200 analyzer (Abbott, Wiesbaden, Germany)	No information available	ICCL‡
**Creatinine** (mmol/l)	Abbott Architect CI 16200 analyzer (Abbott, Wiesbaden, Germany)	8.0 - 26.5	ICCL‡

AAS, Atomic absorption spectroscopy; ELISA, Enzyme-linked Immunosorbent Assay; HPLC, High-performance liquid chromatography; ICP-MS, Inductively-coupled-plasma mass-spectrometry; LC, Liquid chromatography; LC-MS/MS, Liquid chromatography–mass spectrometry/ mass spectrometry; MCV, Mean corpuscular volume;

Ø Abbott Architect CI 16200 analyzer (Abbott, Wiesbaden, Germany)

‡ ICCL, Institute of Clinical Chemistry and Laboratory Diagnostics, University Hospital Jena, Germany; accredited reference laboratory (D-PL-13144-03-00, valid until 05.06.2023)

Table S3. Lifestyle and socio-economic status – NovAL study (n = 72)

	**Data on Lifestyle and socio-economic status**	**Total**	**CG1**	**CG2**	**IG1**	**IG2**	**p-value**
		n=72	n = 19	n=17	n=19	n = 17	
**Marital status**	Married, living together	3	0	0	2	1	**0.025**
	Married, living separately	1	0	0	1	0	
	Not married, living together with partner	32	9	6	10	7	
	Not married, partner lives separately	24	6	4	6	8	
	Single	12	4	7	0	1	
**Graduation**	Primary school leaving certificate	1	0	0	1	0	**0.309**
	Secondary school leaving certificate	2	0	0	1	1	
	University entrance qualification	69	19	17	17	16	
**Completed vocational training**	Yes	14	4	4	3	3	**0.767**
	No	49	13	11	12	13	
	not specified	9	2	2	4	1	
**University degree**	Yes	29	5	8	10	6	**0.462**
	No	41	13	8	9	11	
	Not specified	2	1	1	0	0	
**Number of persons living in the household**	1	17	6	4	3	4	**0.595**
	2	32	7	8	10	7	
	3	14	3	3	3	5	
	4	6	1	1	3	1	
	5	1	1	0	0	0	
	not specified	2	1	1	0	0	
**Household net income (per month)**	< 500 €	10	4	0	5	1	**0.407**
	501-800 €	29	10	7	3	9	
	801-1000 €	5	0	2	2	1	
	1001-1500 €	5	0	3	2	0	
	1501-2000 €	6	0	1	2	3	
	2001-2500 €	4	2	0	0	2	
	2501-3000 €	3	0	1	2	0	
	> 3000 €	9	3	3	2	1	
	not specified	1	0	0	1	0	
**Smoking status**	Yes	10	5	3	1	1	**0.216**
	Former	6	1	2	2	1	
	No	55	13	11	16	15	
	Not specified	1	0	1	0	0	
**Nutritional supplement**	Yes	9	3	3	3	0	**0.238**
	No	62	16	13	16	17	
	Not specified	1	0	1	0	0	
**Food allergy**	Yes	8	2	1	1	4	**0.433**
	No	63	17	15	18	13	
	Not specified	1	0	1	0	0	

CG1 = no defined background diet, CG2 = defined background diet, IG1 = defined background diet + *Chlorella pyrenoidosa* smoothie, IG2 = defined background diet + *Microchloropsis salina* smoothie

Table S4. Energy and nutrient intake of the NovAL study participants in the week before starting the intervention (self-reports, 5 days, n =72)

	**Control group I**					**Control group II**					**Intervention group I**					**Intervention group II**				
**Energy and nutrients**	**n**	**Median**	**/**	**IQR**	**p**	**n**	**Median**	**/**	**IQR**	**p**	**n**	**Median**	**/**	**IQR**	**p**	**n**	**Median**	**/**	**IQR**	**p**
**Energy** (kcal/day)	19	1701	/	1060	a	17	2139	/	367	a	19	2112	/	826	a	17	2123	/	700	a
**Carbohydrate** (g/day)	19	195	/	96	a	17	232	/	45	a	19	240	/	109	a	17	234	/	76	a
**Fiber** (g/day)	19	24	/	15	a	17	23	/	26	a	19	27	/	14	a	17	29	/	14	a
**Protein** (g/day)	19	67	/	32	a	17	80	/	25	a	19	92	/	48	a	17	82	/	25	a
**Fat** (g/day)	19	67	/	45	a	17	80	/	31	a	19	83	/	32	a	17	79	/	30	a
**SFA** (g/day)	19	29	/	19	a	17	28	/	14	a	19	32	/	13	a	17	31	/	8	a
**MUFA** (g/day)	19	22	/	12	a	17	28	/	5	a	19	28	/	14	a	17	27	/	10	a
**PUFA** (g/day)	19	10.0	/	7.5	a	17	13.2	/	8.8	a	19	13.5	/	7.1	a	17	14.5	/	7.4	a
**C18:3n3** (g/day)	19	1.19	/	0.58	a	17	2.25	/	2.42	b	19	1.76	/	1.35	a,b	17	1.75	/	0.69	a,b
**C20:5n3** (g/day)	19	0.02	/	0.26	a	17	0.04	/	0.12	a	19	0.02	/	0.06	a	17	0.06	/	0.17	a
**C22:6n3** (g/day)	19	0.08	/	0.32	a	17	0.08	/	0.19	a	19	0.08	/	0.15	a	17	0.19	/	0.24	a
**Vitamin A** (mg/day)	19	0.36	/	0.19	a	17	0.31	/	0.18	a	19	0.36	/	0.14	a	17	0.38	/	0.21	a
**Vitamin B_1** (mg/day)	19	1.15	/	0.58	a	17	1.49	/	0.82	a	19	1.51	/	0.50	a	17	1.59	/	0.71	a
**Vitamin B_2** (mg/day)	19	1.17	/	0.49	a	17	1.45	/	0.54	a	19	1.45	/	0.67	a	17	1.41	/	0.46	a
**Vitamin B_6** (mg/day)	19	1.53	/	0.85	a	17	1.89	/	0.93	a	19	2.00	/	0.92	a	17	2.11	/	0.77	a
**Vitamin B_{12}** (µg/day)	19	3.37	/	2.58	a	17	3.20	/	1.50	a	19	3.87	/	2.23	a	17	4.04	/	2.33	a
**Vitamin C** (mg/day)	19	118	/	53	a	17	148	/	108	a	19	144	/	120	a	17	132	/	74	a
**Vitamin D** (µg/day)	19	1.99	/	2.22	a	17	1.41	/	1.04	a	19	1.88	/	1.30	a	17	1.93	/	0.87	a
**Vitamin E** (mg/day)	19	10.34	/	6.97	a	17	9.94	/	14.40	a	19	12.85	/	9.28	a	17	14.21	/	6.65	a
**Vitamin K** (mg/day)	19	0.10	/	0.17	a	17	0.25	/	0.25	a	19	0.27	/	0.20	a	17	0.17	/	0.17	a
**Calcium** (mg/day)	19	841	/	423	a	17	1057	/	460	a	19	859	/	426	a	17	854	/	321	a
**Magnesium** (mg/day)	19	329	/	223	a	17	353	/	263	a	19	392	/	160	a	17	426	/	144	a
**Iron** (mg/day)	19	10.17	/	6.54	a	17	13.69	/	8.66	a	19	12.69	/	4.75	a	17	13.09	/	4.29	a
**Iodine** (µg/day)	19	76	/	47	a	17	104	/	53	a	19	87	/	41	a	17	84	/	41	a
**Copper** (mg/day)	19	1.59	/	0.80	a	17	2.06	/	1.36	a	19	2.01	/	0.96	a	17	2.11	/	0.91	a
**Manganese** (mg/day)	19	3.78	/	3.53	a	17	4.16	/	4.82	a	19	5.18	/	2.70	a	17	4.19	/	3.26	a
**Zinc** (mg/day)	19	9.50	/	4.75	a	17	11.19	/	3.48	a	19	11.33	/	5.25	a	17	10.89	/	3.31	a

IQR, interquartile range; MUFA, monounsaturated fatty acids; PUFA, polyunsaturated fatty acids; SFA, saturated fatty acids

Control group I = no defined background diet, control group II = defined background diet, intervention group I = defined background diet + *Chlorella pyrenoidosa* smoothie, intervention group II = defined background diet + *Microchloropsis salina* smoothie

[ab] Different superscript letters indicate significant differences between the groups ($p<0.05$)

Table S5. Anthropometric data, body composition and blood pressure of the NovAL study participants at the last blood drawing (n =72)

		Control group I					Control group II					Intervention group I					Intervention group II				
	**Weeks**	**n**	**Median**	**/**	**IQR**	**p**	**n**	**Median**	**/**	**IQR**	**p**	**n**	**Median**	**/**	**IQR**	**p**	**n**	**Median**	**/**	**IQR**	**p**
Anthropometric Data																					
**Age** (yrs)		19	22.0	/	5.5	a	17	24.0	/	6.0	a	19	24.0	/	5.0	a	17	23.0	/	5.0	a
**Height** (m)		19	1.71	/	0.13	a	17	1.74	/	0.10	a	19	1.68	/	0.13	a	17	1.71	/	0.14	a
**Weight**	0	19	62.35	/	14.83	a	17	64.85	/	9.95	a	19	66.95	/	18.93	a	17	66.30	/	14.50	a
(kg)	2	19	63.30	/	14.55	a	17	64.40	/	11.20	a	19	66.85	/	17.95	a	17	65.35	/	14.95	a
	Δ	19	0.00	/	0.75	a	17	-0.85	/	1.10	a	19	-0.45	/	1.25	a	17	-0.65	/	1.10	a
**BMI**	0	19	21.63	/	2.98	a	17	21.80	/	1.70	a	19	22.49	/	3.31	a	17	23.16	/	4.08	a
(kg/m^2)	2	19	21.55	/	3.33	a	17	21.52	/	1.98	a	19	22.29	/	3.18	a	17	23.16	/	4.08	a
	Δ	19	0.00	/	0.25	a	17	-0.28	/	0.37	a	19	-0.15	/	0.37	a	17	-0.18	/	0.37	a
Body composition																					
**Total energy expenditure**	0	19	2028	/	540	a	17	2138	/	457	a	19	2098	/	382	a	17	2015	/	476	a
(kcal/day)	2	19	2019	/	535	a	17	2135	/	458	a	19	2067	/	420	a	17	2015	/	409	a
	Δ	19	0	/	6	a	17	-6	/	9	a	19	-3	/	12	a	17	-5	/	8	a
**Resting energy**	0	19	1435	/	357	a	17	1455	/	276	a	19	1449	/	330	a	17	1439	/	340	a
**expenditure**(kcal/day)	2	19	1435	/	355	a	17	1449	/	274	a	19	1449	/	326	a	17	1439	/	292	a
	Δ	19	0	/	4	a	17	-5	/	7	a	19	-3	/	7	a	17	-3	/	6	a
**Relative fat mass**	0	19	26.44	/	6.54	a	17	28.63	/	21.06	a	19	26.07	/	11.19	a	17	24.73	/	10.34	a
(%)	2	19	25.25	/	5.40	a	17	26.28	/	18.66	a	19	25.47	/	12.14	a	17	24.69	/	10.54	a
	Δ	19	-0.28	/	1.18	a	17	-0.35	/	2.03	a	19	-0.64	/	1.43	a	17	-0.78	/	0.99	a
**Absolute fat mass**	0	19	15.10	/	6.93	a	17	16.76	/	9.81	a	19	15.26	/	9.05	a	17	15.00	/	7.30	a
(kg)	2	19	14.97	/	6.49	a	17	16.01	/	8.13	a	19	14.52	/	10.07	a	17	14.73	/	7.83	a
	Δ	19	-0.19	/	0.96	a	17	-0.52	/	1.23	a	19	-0.58	/	0.94	a	17	-0.62	/	0.67	a
**Fat free mass**	0	19	44.39	/	16.12	a	17	46.20	/	16.98	a	19	49.20	/	15.85	a	17	47.28	/	13.30	a
(kg)	2	19	45.10	/	15.19	a	17	44.89	/	17.23	a	19	48.24	/	15.89	a	17	46.61	/	12.55	a
	Δ	19	0.13	/	1.32	a	17	-0.02	/	1.35	a	19	0.18	/	0.98	a	17	-0.14	/	0.87	a
**Fat free mass index**	0	19	15.57	/	2.88	a	17	15.47	/	3.29	a	19	17.33	/	1.37	a	17	17.16	/	2.13	a
(kg/m^2)	2	19	15.73	/	2.49	a	17	15.37	/	3.39	a	19	17.38	/	1.87	a	17	16.99	/	2.90	a
	Δ	19	0.03	/	0.47	a	17	-0.01	/	0.48	a	19	0.06	/	0.38	a	17	-0.06	/	0.28	a

**Fat mass indices**	0	19	5.81 / 2.19	a	17	5.66 / 4.79	a	19	4.98 / 3.04	a	17	5.25 / 3.30	a
(kg/m^2)	2	19	5.60 / 1.89	a	17	5.29 / 4.06	a	19	4.90 / 2.92	a	17	5.19 / 3.02	a
	Δ	19	-0.07 / 0.34	a	17	-0.19 / 0.40	a	19	-0.21 / 0.31	a	17	-0.22 / 0.23	a
**Visceral adipose tissue**	0	19	0.36 / 0.47	a	17	0.55 / 0.31	a	19	0.56 / 1.03	a	17	0.25 / 0.64	a
(l)	2	19	0.33 / 0.18	a	17	0.51 / 0.38	a	19	0.46 / 0.64	a	17	0.24 / 0.60	a
	Δ	19	-0.09 / 0.20	a	17	-0.08 / 0.17	a	19	-0.11 / 0.23	a	17	-0.09 / 0.22	a
**Total body water**	0	19	33.28 / 11.29	a	17	33.98 / 11.98	a	19	36.39 / 11.25	a	17	34.9 / 9.29	a
(l)	2	19	33.58 / 10.29	a	17	33.08 / 11.65	a	19	35.35 / 11.25	a	17	34.09 / 8.48	a
	Δ	19	0.01 / 0.98	a	17	0.03 / 1.39	a	19	-0.01 / 0.79	a	17	-0.06 / 0.80	a
**Extracellular water**	0	19	13.91 / 3.90	a	17	15.17 / 4.47	a	19	15.17 / 3.84	a	17	15.01 / 2.73	a
(l)	2	19	14.33 / 3.64	a	17	14.55 / 4.37	a	19	14.98 / 3.95	a	17	14.91 / 3.17	a
	Δ	19	-0.11 / 0.54	a	17	-0.20 / 0.83	a	19	-0.02 / 0.33	a	17	-0.22 / 0.46	a
**transcellular water**	0	19	41.81 / 2.76	a	17	42.08 / 2.57	a	19	41.78 / 3.95	a	17	42.99 / 2.45	a
(l)	2	19	42.12 / 3.17	a	17	41.62 / 2.34	a	19	41.69 / 3.76	a	17	42.79 / 2.60	a
	Δ	19	0.16 / 0.41	a	17	0.05 / 1.24	a	19	0.06 / 0.64	a	17	-0.47 / 1.04	a
**Skeletal muscle mass**	0	19	21.27 / 9.06	a	17	21.12 / 9.03	a	19	21.48 / 8.93	a	17	21.97 / 7.65	a
(kg)	2	19	20.99 / 8.54	a	17	20.65 / 9.59	a	19	21.72 / 9.12	a	17	20.85 / 7.35	a
	Δ	19	-0.26 / 0.81	a	17	-0.32 / 1.28	a	19	-0.24 / 0.77	a	17	-0.07 / 0.87	a
Blood pressure													
**Systole**	0	19	127 / 17	a	17	121 / 13	a	19	118 / 16	a	17	125 / 12	a
(mmHg)	2	19	121 / 13	a	17	115 / 11	a	19	116 / 18	a	17	117 / 11	a
	Δ	19	-10 / 16	a	17	-6 / 13	a	19	-5 / 13	a	17	-4 / 17	a
**Diastole**	0	19	88 / 8	a	17	85 / 11	a	19	86 / 11	a	17	86 / 4	a
(mmHg)	2	19	84 / 11	a	17	83 / 9	a	19	85 / 12	a	17	82 / 8	a
	Δ	19	-5 / 19	a	17	-3 / 8	a	19	-4 / 10	a	17	-7 / 6	a
**Pulse**	0	19	70 / 20	a	17	68 / 21	a	19	72 / 21	a	17	67 / 12	a
(bpm)	2	19	68 / 13	a	17	69 / 19	a	19	69 / 10	a	17	66 / 5	a
	Δ	19	3 / 16	a	17	0 / 9	a	19	1 / 9	a	17	2 / 10	a

BMI, body mass index; IQR, interquartile range

Control group I = no defined background diet, control group II = defined background diet, intervention group I = defined background diet + *Chlorella pyrenoidosa* smoothie, intervention group II = defined background diet + *Microchloropsis salina* smoothie

Δ, difference between parameter baseline and end of the 2 week

[a] Different superscript letters indicate significant differences between the groups ($p<0.05$)

* Significant differences between start and the end of the study period ($p<0.05$)

Table S6. Further blood parameters of the NovAL study participants (n = 72)

		Control group I						Control group II						Intervention group I						Intervention group II					
	**Weeks**	**n**	**Median**	**/**	**IQR**	**p**		**n**	**Median**	**/**	**IQR**	**p**		**n**	**Median**	**/**	**IQR**	**p**		**n**	**Median**	**/**	**IQR**	**p**	
**Relative basophil granulocytes** (%)	0	19	1	/	0	a		17	1	/	0	a		19	1	/	0	a		17	1	/	0	a	
	2	19	1	/	0	a		17	1	/	0	a		19	1	/	0	a		17	1	/	1	a	
	Δ	19	0	/	0	a		17	0	/	0	a		19	0	/	1	a		17	0	/	0	a	
**Absolute basophil granulocytes** (Gpt/l)	0	19	0.05	/	0.02	a		17	0.04	/	0.02	a		19	0.05	/	0.03	a		17	0.05	/	0.02	a	
	2	19	0.04	/	0.02	a		17	0.05	/	0.03	a		19	0.04	/	0.02	a		17	0.04	/	0.02	a	
	Δ	19	0.00	/	0.01	a		17	0.00	/	0.02	a		19	-0.01	/	0.01	a		17	-0.01	/	0.02	a	
**Relative eosinophil granulocytes** (%)	0	19	2	/	2	a		17	2	/	2	a		19	2	/	1	a		17	2	/	3	a	
	2	19	3	/	3	a		17	2	/	3	a		19	4	/	2	a		17	3	/	3	a	
	Δ	19	0	/	1	a		17	0	/	1	a		19	1	/	2	a		17	1	/	2	a	
**Absolute eosinophil granulocytes** (Gpt/l)	0	19	0.14	/	0.14	a		17	0.11	/	0.12	a		19	0.13	/	0.13	a		17	0.09	/	0.15	a	
	2	19	0.14	/	0.16	a		17	0.12	/	0.08	a		19	0.18	/	0.15	a		17	0.18	/	0.14	a	
	Δ	19	0.01	/	0.03	a		17	0.01	/	0.03	a		19	0.05	/	0.08	a		17	0.08	/	0.07	a	
**Relative neutrophile granulocytes** (%)	0	19	54	/	11	a		17	56	/	13	a		19	54	/	10	a		17	58	/	13	a	
	2	19	54	/	14	a		17	55	/	14	a		19	60	/	8	a		17	58	/	12	a	
	Δ	19	-4	/	12	a		17	-1	/	6	a		19	4	/	8	a		17	0	/	12	a	
**Absolute neutrophile granulocytes** (Gpt/l)	0	19	3.34	/	1.85	a		17	3.02	/	1.25	a		19	2.91	/	1.32	a		17	2.52	/	1.68	a	
	2	19	2.83	/	1.21	a		17	3.11	/	1.27	a		19	3.69	/	1.44	a		17	3.34	/	1.59	a	
	Δ	19	-0.64	/	1.96	a		17	-0.03	/	1.06	a		19	0.55	/	0.87	a		17	-0.04	/	1.25	a	
**Relative lymphocytes** (%)	0	19	32	/	8	a		17	34	/	12	a		19	36	/	11	a		17	31	/	10	a	
	2	19	32	/	9	a		17	32	/	9	a		19	29	/	9	a		17	29	/	9	a	
	Δ	19	3	/	11	a		17	-1	/	7	a		19	-4	/	5	a		17	-1	/	8	a	
**Absolute lymphocytes** (Gpt/l)	0	19	1.77	/	0.31	a		17	1.73	/	0.57	a		19	1.72	/	0.57	a		17	1.64	/	0.46	a	
	2	19	1.84	/	0.46	a		17	1.94	/	0.63	a		19	1.71	/	0.51	a		17	1.72	/	0.43	a	
	Δ	19	0.08	/	0.31	a		17	0.11	/	0.48	a		19	-0.04	/	0.29	a		17	-0.01	/	0.27	a	
**Relative monocytes** (%)	0	19	8	/	4.5	a		17	9	/	2	a		19	8	/	2	a		17	8	/	2	a	
	2	19	7	/	4	a		17	9	/	3	a		19	8	/	2	a		17	9	/	3	a	
	Δ	19	1	/	2	a		17	1	/	1	a		19	0	/	2	a		17	1	/	1	a	

**Absolute monocytes**	0	19	0.44 / 0.22	a	17	0.42 / 0.21	a	19	0.39 / 0.20	a	17	0.43 / 0.10	a
(Gpt/l)	2	19	0.44 / 0.22	a	17	0.54 / 0.16	a	19	0.46 / 0.20	a	17	0.44 / 0.16	a
	Δ	19	0.01 / 0.13	a	17	0.04 / 0.11	a	19	0.03 / 0.08	a	17	0.04 / 0.17	a
**Erythrocytes**	0	19	4.8 / 0.5	a	17	4.7 / 0.7	a	19	4.9 / 0.5	a	17	4.5 / 0.7	a
(g/l)	2	19	4.6 / 0.6	a	17	4.7 / 0.6	a	19	4.6 / 0.8	a	17	4.5 / 0.4	a
	Δ	19	-0.1 / 0.2	a	17	0.0 / 0.3	a	19	-0.1 / 0.4	a	17	0.0 / 0.3	a
**Leukocytes**	0	19	6.1 / 2.1	a	17	5.3 / 1.6	a	19	5.6 / 1.7	a	17	5.7 / 2.9	a
(Gpt/l)	2	19	5.6 / 1.0	a	17	5.5 / 1.2	a	19	6.3 / 1.8	a	17	5.5 / 1.6	a
	Δ	19	-0.7 / 1.8	a	17	0.1 / 0.9	a	19	0.8 / 1.3	a	17	0.1 / 1.5	a
**Thrombocytes**	0	19	263 / 42	a	17	232 / 99	a	19	243 / 41	a	17	211 / 40	a
(Gpt/l)	2	19	281 / 47	a	17	239 / 74	a	19	233 / 51	a	17	234 / 55	a
	Δ	19	-2 / 36	a	17	3 / 31	a	19	-5 / 24	a	17	13 / 30	a

IQR, interquartile range

Control group I = no defined background diet, control group II = defined background diet, intervention group I = defined background diet + *Chlorella pyrenoidosa* smoothie, intervention group II = defined background diet + *Microchloropsis salina* smoothie

Δ, difference between parameter baseline and end of the 2 week

[a] Different superscript letters indicate significant differences between the groups ($p<0.05$)

* Significant differences between start and the end of the study period ($p<0.05$)

Socio-economic data and lifestyle

Significant differences were seen in marital status of all four groups ($p \leq 0.05$, Table S3). While the participants of the control group I (CG1), intervention group I (IG1) and intervention group II (IG2) were mainly in a relationship living with or without their partner, more than one third of the participants in control group II (CG2) were singles (Table S3). Only 3 out of 72 participants of the NovAL study had no university entrance qualification (Table S3). A completed vocational training had 3-4 participants in each group, but the majority had not completed vocational training (Table S3). More than half of the NovAL study population had no university degree. The participants in CG1 and IG2 had mostly a university degree while in the CG2 and IG1 were balanced between with and without university degree (Table S3). Yet, there was no significant differences. Most of the subjects lived in a household with one to three persons (Table S3). The household net income per month was in all groups mostly under 800 € (Table S3). Active smokers were with 7 % generally low in the NovAL study cohort. CG1 had with five participants the highest amount of active smokers while group II included three smokers and IG1 and IG2 had only 1 smoker (Table S3). Three months prior to the study start, three participants of each group, except IG2 with no person, consumed nutritional supplements (Table S3). 63 participants declared no food allergies. IG2 had with four persons the most participants with food allergies while in the remaining groups one to two people declared allergic reactions to specific foods (Table S3).
